# Transhydrogenase Promotes the Robustness and Evolvability of *E. coli* Deficient in NADPH Production

**DOI:** 10.1371/journal.pgen.1005007

**Published:** 2015-02-25

**Authors:** Hsin-Hung Chou, Christopher J. Marx, Uwe Sauer

**Affiliations:** 1 Institute of Molecular Systems Biology, ETH Zürich, Zürich, Switzerland; 2 Department of Biochemistry, University of Cambridge, Cambridge, United Kingdom; 3 Department of Biological Sciences, University of Idaho, Moscow, Idaho, United States of America; University of Houston, UNITED STATES

## Abstract

Metabolic networks revolve around few metabolites recognized by diverse enzymes and involved in myriad reactions. Though hub metabolites are considered as stepping stones to facilitate the evolutionary expansion of biochemical pathways, changes in their production or consumption often impair cellular physiology through their system-wide connections. How does metabolism endure perturbations brought immediately by pathway modification and restore hub homeostasis in the long run? To address this question we studied laboratory evolution of pathway-engineered *Escherichia coli* that underproduces the redox cofactor NADPH on glucose. Literature suggests multiple possibilities to restore NADPH homeostasis. Surprisingly, genetic dissection of isolates from our twelve evolved populations revealed merely two solutions: (1) modulating the expression of membrane-bound transhydrogenase (mTH) in every population; (2) simultaneously consuming glucose with acetate, an unfavored byproduct normally excreted during glucose catabolism, in two subpopulations. Notably, mTH displays broad phylogenetic distribution and has also played a predominant role in laboratory evolution of *Methylobacterium extorquens* deficient in NADPH production. Convergent evolution of two phylogenetically and metabolically distinct species suggests mTH as a conserved buffering mechanism that promotes the robustness and evolvability of metabolism. Moreover, adaptive diversification via evolving dual substrate consumption highlights the flexibility of physiological systems to exploit ecological opportunities.

## Author Summary

The structure of biological networks, like traffic systems or the Internet, features few hubs connected by numerous components. Though the conservation and high connectivity of hubs serve as key junctions to promote network expansion, addition or removal of connections surrounding hubs may disturb the whole system through their global linkage. How do biological networks mitigate hub perturbations during evolution? Using metabolism as an example, we studied the physiological and evolutionary consequences of genetically perturbed production of a hub metabolite NADPH in *E*. *coli*. We found that the expression of mTH, a phylogenetically conserved enzyme, was immediately upregulated and essential to counteract the hub perturbation. Moreover, long-term evolution of this pathway-modified *E*. *coli* in glucose growth media recurrently selected for mTH-upregulating mutations to restore the NADPH balance in all twelve replicate populations, regardless of several alternative solutions suggested in the literature. Corroborated by similar findings from laboratory evolution of a highly diverged species *M*. *extorquens*, our study suggests that mechanisms dedicated to mitigating hub perturbations promote both the robustness and evolvability of biological networks.

## Introduction

Metabolic networks, consisting of metabolites connected through biochemical reactions, are central to life by extracting energy from nutrients and converting chemicals into building blocks of organisms. Similar to the architecture of the Internet and other biological networks (e.g. gene regulation, protein interactomes), the connectivity of metabolism is skewed by few metabolites (e.g. ATP, glutamate, NADH, NADPH) participating in myriads of reactions [1,2]. These hub metabolites are phylogenetically conserved, recognized by diverse enzymes, and are proposed to be stepping stones for the evolutionary expansion of enzyme families and biochemical pathways [3–5]. Though chemically similar, redox cofactors NADH and NADPH function as distinct electron carriers in over 70 and 50 redox reactions in *Escherichia coli*, respectively [3]. While NADH is consumed primarily in respiration to generate ATP and the proton motive force, NADPH provides the reducing power to synthesize a variety of biomolecules. The catabolic production of each redox cofactor must be deliberately adjusted to match the anabolic demand for cell growth. This delicate balance, however, is disrupted when organisms experience oxidative stress [6,7], switch substrates or growth conditions [8,9], or evolve pathways that alter the NAD(H)/NADP(H) production or consumption [10–12]. Consequently, mechanisms that safeguard the balance of redox currencies, or hub metabolites in general, may not only confer physiological robustness to survive environmental fluctuations but also promote the flexibility of metabolism to accommodate mutations that alter its network structure (evolvability) [13].

**Fig 1:**
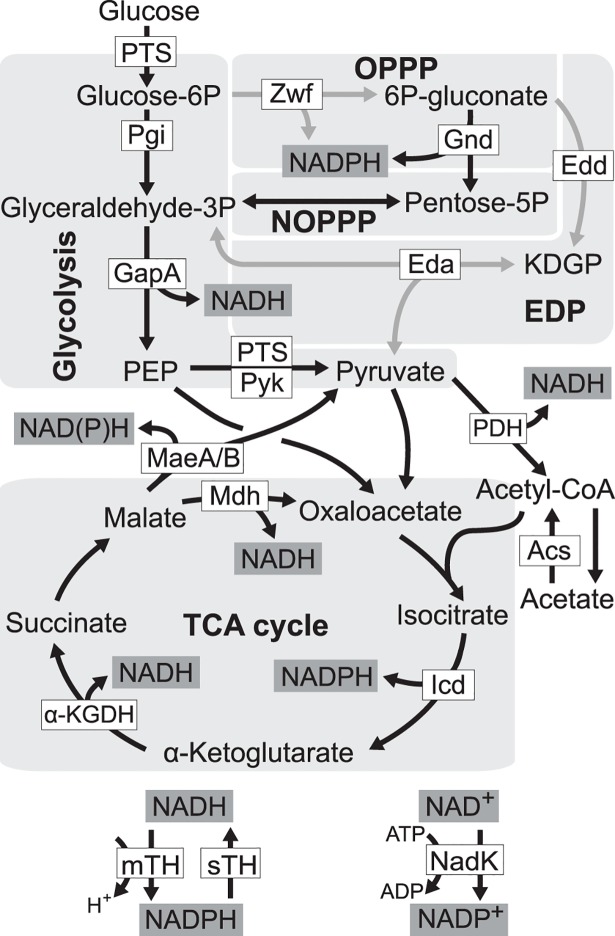
Redox cofactor production during growth on glucose. Five pathways in central metabolism of *E*. *coli*, glycolysis, oxidative pentose phosphate pathway (OPPP), non-oxidative pentose phosphate pathway (NOPPP), Entner-Doudoroff pathway (EDP), and tricarboxylic acid (TCA) cycle, are highlighted by light grey. Reactions disrupted in *E*. *coli* ZED are shown as grey arrow lines. PTS, glucose phosphotransferase system; Zwf, glucose-6-phosphate dehydrogenase; Gnd, 6-phosphogluconate dehydrogenase; Pgi, phosphoglucose isomerase; Edd, 6-phosphogluconate dehydrogenase; KDGP, 2-keto-3-deoxy-6-phosphogluconate; Eda, KDGP aldolase; GapA, glyceraldehyde-3-phosphate dehydrogenases; Pyk, pyruvate kinase; PDH, pyruvate dehydrogenase; MaeA/B, NAD- and NADP-dependent malic enzymes; Mdh, malate dehydrogenase; Acs, acetyl-CoA synthetase; Icd, isocitrate dehydrogenase; α-KGDH, α-ketoglutarate dehydrogenase; mTH, membrane-bound transhydrogenase; sTH, soluble transhydrogenase; NadK, NAD kinase; PEP, phosphoenolpyruvate.

Based on network analysis and physiological characterization, a number of mechanisms have been proposed to mediate redox cofactor levels in different species, including differential expression of isoenzymes utilizing different cofactors, modulating the activity of NAD(H) kinase, converting the cofactor specificity of catabolic enzymes, rerouting metabolic flux, or enhancing hydride transfer reactions between NADH and NADPH (NADH + NADP^+^ ↔ NAD^+^ + NADPH) catalyzed by membrane-bound transhydrogenase (mTH, forward reaction) and soluble transhydrogenase (sTH, reverse reaction) [14–18] (Fig. 1). It remains unclear which of these mechanisms is more likely to participate in pathway evolution to mitigate the adverse impact caused by changes in the network structure and redox cofactor stoichiometry.

We probed this question by genetically dissecting laboratory evolution of *E*. *coli* deficient in NADPH production. During growth on glucose *E*. *coli* wild-type (WT) generates NADPH through glucose-6-phosphate dehydrogenase and 6-phosphogluconate dehydrogenase in the oxidative pentose phosphate (OPP) pathway, mTH, isocitrate dehydrogenase in the tricarboxylic acid (TCA) cycle, and to a smaller extent, NADP-dependent malic enzyme [19] (Fig. 1). Previously, genes of the OPP pathway (*zwf*, encoding glucose-6-phosphate dehydrogenase) and the Entner-Doudoroff pathway (*edd* and *eda* encoding 6-phosphogluconate dehydrogenase, and 2-keto-3-deoxy-6-phosphogluconate aldolase, respectively) were deleted to generate a low NADPH-producing *E*. *coli* strain ZED (Δ*z* *wf* Δ*ed* *d* Δ*ed* *a*). While disruption of the Entner-Doudoroff pathway yielded no growth phenotype, blockage of the OPP pathway caused a 15% decrease in growth rates on glucose [19,20]. We used each of *E*. *coli* WT and *E*. *coli* ZED to establish twelve independent populations evolved under identical growth conditions over a thousand generations. By comparing the evolution of two strains differing in a primary NADPH-generating pathway, we revealed the genetic differentiation following pathway modification, uncovered adaptive diversification driven by the NADPH shortage, and identified mTH as the predominant redox balancing strategy that promoted both the robustness and evolvability of central metabolism.

## Results

### Adaptation of *E*. *coli* ZED resulted in higher phenotypic divergence than WT adaptation

**Fig 2:**
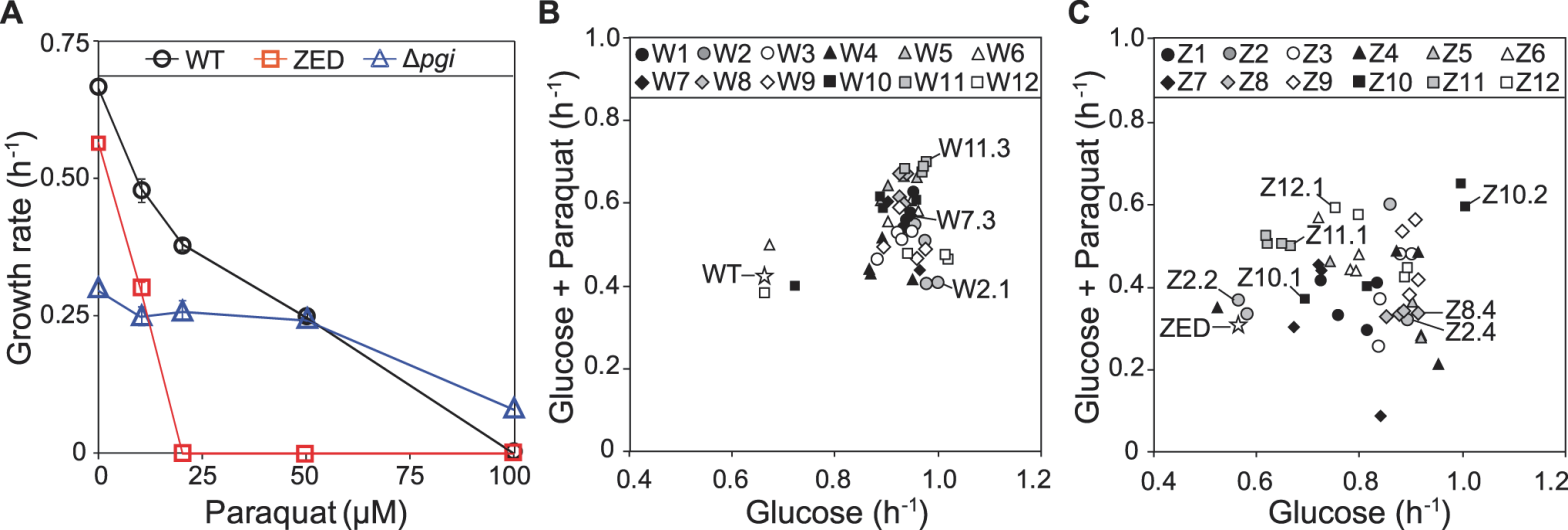
Growth rates of *E*. *coli* in M9 glucose medium. (A) Sensitivity of *E*. *coli* WT, ZED, and Δ*pgi* strains to paraquat. (B) Growth of *E*. *coli* WT and W isolates in response to 10 μM paraquat. (C) Growth of *E*. *coli* ZED and Z isolates in response to 10 μM paraquat. Evolved isolates selected for genome sequencing are indicated in (B) and (C). Error bars indicate 95% confidence intervals (C.I.) based on four independent measurements.

We evolved *E*. *coli* WT (MG1655) and the low NADPH-producing *E*. *coli* ZED that catabolized glucose exclusively through glycolysis. Using each strain we established twelve replicate populations (termed W1-W12 and Z1-Z12, respectively) grown in M9 glucose batch culture over 113 passages (equivalent to 1017 cell generations; see Materials and Methods). Evolution of *E*. *coli* WT represents a control to help identify adaptation specific to the suboptimal NADPH production of the ZED strain. The rate of adaptation of W and Z populations over one thousand generations both decelerated, as typically seen in experimental evolution (S1A Fig., S1B Fig.) [10,21]. Relative to their ancestors, the Z populations showed larger growth improvements. Yet in terms of growth rates measured as a whole population, both the twelve W and the twelve Z replicate populations reached a similar range (0.8–1.1 h^-1^) at the end of evolution experiments (one-way ANOVA, *P* = 0.309). Growth rates of four isolates from each of the end-point W and Z populations were quantified (Fig. 2B, Fig. 2C). These isolates were chosen based on unique colony morphology on M9 glucose agar in order to enrich the discovery of phenotypic diversity (S1 Table). Although this sampling procedure was nonrandom, growth rates of handpicked individuals correlated significantly with the growth rates of the whole populations on glucose (Pearson’s r = 0.641, *P* = 0.025; Spearman’s ρ = 0.650, *P* = 0.022; S1C Fig.), suggesting that sampling bias was a minor concern. Besides growth in M9 glucose minimal medium, these isolates were also tested for their resistance to the oxidizing agent, paraquat (Fig. 2A). This extra screening appeared to correlate with the ability of isolates to produce NADPH, as the ZED ancestor was hypersensitive to paraquat compared to WT, while the NADPH-overproducing *E*. *coli* Δ*pgi* (encoding phosphoglucose isomerase) [19] exhibited higher tolerance.

Adaptation of *E*. *coli* WT and ZED under identical environments resulted in distinctive phenotypic outcomes (Fig. 2B, Fig. 2C). Relative to evolved isolates from the W populations, Z isolates on average attained lower growth rates on glucose (0.92 and 0.81, respectively; one-way nested ANOVA, *P* = 1.5e-4). While each of the W replicate populations evolved similar growth rates (one-way ANOVA, *P* = 0.576), Z replicate populations exhibited significant heterogeneity (one-way ANOVA, *P* = 0.011). Notably, within each of the three Z populations, Z2, Z4, and Z10 (coefficients of variation as 23.4%, 24.2%, and 17.1%, respectively), we discovered isolates exhibiting distinct growth rates on glucose, which were termed slow-growing (SG) and fast-growing (FG) isolates accordingly. Moreover, evolution of Z populations in M9 glucose medium led to differentiation in diauxic growth (S1 Table). During growth in glucose-fed batch culture *E*. *coli* typically goes through three sequential stages before reaching the stationary phase: (1) growth through catabolizing glucose and simultaneously secreting acetate until the depletion of glucose; (2) physiological acclimation in preparation for switching growth substrates (i.e. diauxic shift); (3) growth on acetate. While this characteristic pattern was observed in both WT and ZED and retained in all 48 W isolates, 12 out of 48 Z isolates exhibited just a single growth phase (see Materials and Methods for the detection of diauxic growth). Such phenotypic divergence between W and Z populations was statistically significant (Fisher’s exact test, *P* = 1.12e-4). Notably, the twelve Z isolates without diauxic growth tended to grow more slowly on glucose than the rest of Z isolates (0.66 and 0.86, respectively; one-way nested ANOVA, *P* = 4.39e-4), indicating higher phenotypic diversification and a possibility of ecological differentiation within the Z populations.

### Genome sequencing revealed common and unique genetic bases underlying the adaptation of *E*. *coli* WT and ZED

To elucidate the genetic bases of phenotypic divergence between W and Z populations, we sequenced the genomes of three W isolates (W2.1, W7.3, W11.3) and seven Z isolates that spanned the phenotypic distribution (Fig. 2B, Fig. 2C). Among the seven Z isolates, three (Z8.4, Z11.1, Z12.1) were from apparently homogenous populations where individuals exhibited similar growth phenotypes, while the remaining four came from two heterogeneous populations where FG isolates (Z2.4, Z10.2) coexisted with SG isolates without diauxic growth (Z2.2, Z10.1). Additionally, we sequenced our lab stocks *E*. *coli* WT and *E*. *coli* ZED in order to identify potential genetic differences relative to the published genome sequence of *E*. *coli* MG1655 (GenBank accession no. U00096.3) [22]. Sequencing by the Illumina HiSeq 2000 system generated 100 bp paired-end reads with 450- to 700-fold average coverage across the genomes of the twelve sequenced strains (ENA accession no. PRJEB5802), thus allowing accurate identification of genetic variations. Genome sequencing of our ancestral *E*. *coli* WT and *E*. *coli* ZED revealed 15 genetic differences relative to the reference genome (S2 Table), some of which have been reported in other *E*. *coli* MG1655 stocks [23,24]. Aside from these, we found a total of 13 and 31 mutations in the three W isolates and the seven Z isolates, respectively (Table 1). Each isolate acquired 2 to 7 mutations. These mutations consisted of 22 point mutations (50%), 9 small (≤ 100 bp) insertions/deletions (indel, 20.5%), 9 large (> 100 bp) indels (20.5%, S2 Fig.), and 4 transpositions of insertion sequences (IS, 9.1%). In accord with earlier studies of bacterial mutations [25,26], point mutations revealed here (15 of 22) often led to G/C→A/T substitutions (also known as AT mutational bias [26]). Moreover, 16 of the 17 point mutations in coding regions caused nonsynonymous substitutions, a bias suggesting that many of these mutations were adaptive.

**Table 1:** Mutations in evolved isolates derived from *E*. *coli* WT and *E*. *coli* ZED.

Gene or region	W2.1	W7.3	W11.3	Z2.2	Z2.4	Z8.4	Z10.1	Z10.2	Z11.1	Z12.1
*Crp*									H20Y	
*cyaA*						A246V			D300E	
*'eptB-yifN'*								250 kb Ampl.^b^		
*fadR*							N166D			
*fimA*	::IS*186* (502/503)									
*ftsW*	Δ1bp (1199)									
*higB*								Δ18 bp (141–158)		
*hupA*						c→t (-46)				Δ2 bp (67–68)
*isrC*								g→t (-7)		
*kdsD*	1 bp Ins. (g, 737/738)									
*ligA*										g→a (1518)
*pntAB*					t→c (-6)			39 kb Ampl.^c^		t→c (-6)
*ppiB*							H42L			
*proQ*							L91Q			
*ptsA*								128 kb Ampl.^d^		
*ptsG*				G295V			Δ10 bp (189–198)			
*ptsI*										R304C
*pykF/lpp*	c→a (+34/-277)	::IS*2* (+20/-291)								
*pyrE*			Δ1bp (-33)			Δ1bp (-33)				
*rhsB-rhsA*	139 kb Ampl.^a^	139 kb Ampl.			139 kb Ampl.	139 kb Ampl.			139 kb Ampl.	
*Rph*					Δ82bp (611–692)					
*rpoB*		G744S								S638R
*rpoC*			V801F							S263F
*rpoD*		D213Y		D213Y						
*rpoS*										E76K
*rpsB*									Δ98 bp (677–774)	
*'yaiT-yaiC'*									12 kb Del.^e^	
*yeaG*									::IS*5* (-95/-96)	
*yeaH*			::IS*2* (990/991)							
*yjhU*			I164S							

^a^Two- to three-fold amplification of the 139 kb region likely resulted from homologous recombination between two recombination hot spots, *rhsA and rhsB*, surrounding the amplified region.

^b^Two-fold amplification of the 250 kb region resulted from two novel IS*2* transpositions into *eptB* (783/784) and a pseudogene *yifN'* (359/360) followed by the duplication of the IS*2-'eptB-yifN'*-IS*2* fragment.

^c^Four-fold amplification of a 39 kb region encompassing the *pntAB* operon resulted from a novel IS*2* transposition into *ydgA* (211/212) followed by homologous recombination with IS*2E* upstream of *rspB* to generate tandem duplication of the IS*2E-rspB-ydgA'-*IS*2* fragment.

^d^Two-fold amplification of a 128 kb region (*rrfA-gltT*) encompassing *ptsA* likely resulted from homologous recombination between two 23S rRNA genes, *rrlA and rrlB*, surrounding the amplified region.

^e^The 12 kb deletion resulted from a novel IS*3* transposition into *yaiC* (803/804) followed by homologous recombination with IS*3B* upstream of *'yaiT* to remove the *'yaiT-yaiC'* region.

Comparison of sequenced genomes identified mutations shared between W and Z isolates, likely associated with adaptation to general growth conditions. For example, nonsynonymous substitutions in genes encoding the RNA polymerase subunits (RpoB, RpoC, RpoD) may reprogram the transcriptional network to promote growth in the M9 minimal medium [27]. Mutations in the *pyrE* gene (encoding orotate phosphoribosyltransferase) and the upstream *rph* gene (encoding 16S rRNA ribonuclease) may improve the inefficient pyrimidine biosynthesis of *E*. *coli* MG1655 in the minimal medium [28]. The exact role of parallel amplification of the 139 kb region between the *rhsB* and *rhsA* genes was unclear (S2A Fig.) as it contained genes involved in protein synthesis, tRNA synthesis, sugar metabolism, and intercellular growth inhibition and several genes of unknown function [29]. This 139 kb tandem duplication resulted from unequal crossover between 3.7 kb homologous regions of *rhsB* and *rhsA* genes and has been frequently observed in *E*. *coli* [30].

Although only three W isolates were sequenced to help distinguish mutations specific to *E*. *coli* ZED, this limited sampling uncovered a key gene unique to the adaptation of *E*. *coli* WT with the intact OPP pathway. Two isolates W2.1 and W7.3 independently acquired mutations right downstream of the pyruvate kinase gene (*pykF*) (Table 1). Pyruvate kinase controls flux through the lower part of glycolysis and indirectly affect glucose uptake through modulating the concentration of phosphoenolpyruvate, a substrate competed by pyruvate kinase and the glucose phosphotransferase system (PTS) (Fig. 1) [31]. *pykF* mutations have also emerged repeatedly in long-term evolution of the *E*. *coli* B strain under similar environments [32]. The recurrence of *pykF* mutations in OPP pathway-containing W isolates and *E*. *coli* B underscores the impact of pathway structure on determining metabolic evolution, despite the substantial genetic divergence between *E*. *coli* MG1655 and *E*. *coli* B [33]. On the contrary, examination of the *pykF* loci of representative Z isolates by genome sequencing (Z2.2, Z2.4, Z8.4, Z10.1, Z10.2, Z11.1, Z12.1) and Sanger sequencing (Z1.1, Z3.3, Z4.3, Z5.1, Z6.1, Z7.2, Z9.1) did not reveal any mutations.

### Adaptation of *E*. *coli* ZED entailed genes encoding the mTH, the cAMP-CRP regulation, and the PTS system

Among mutations unique to Z isolates, we found enrichment in three functional modules (Table 1). These include three mutations in mTH (encoded by the *pntAB* operon) that transfers hydrides from NADH to NADP^+^, four mutations in constituents of the PTS system (encoded by *ptsG*, *ptsI*, and *ptsA*), and three mutations in the adenylate cyclase (encoded by *cyaA*) and the cAMP receptor protein (CRP, encoded by *crp*) that forms the cAMP-CRP regulation. Surprisingly, besides the mTH mutations, none of the others have a clear role in modulating the production and consumption of redox cofactors. We validated the absence of Z-specific mutations in W isolates by sequencing the *pntAB* and *cyaA* loci of nine W isolates (W1.1, W3.2, W4.3, W5.4, W6.1, W8.4, W9.2, W10.1, W12.2) aside from three genome-sequenced W isolates (W2.1, W7.3, W11.3). Mutations in components of cAMP-CRP and PTS may be functionally related. Upon the depletion of glucose, the phosphorylated EIIA component of PTS is known to allosterically activate the adenylate cyclase and promote the production of cAMP, an allosteric effector required for the binding of CRP to specific DNA sequences [31]. Increased formation of the cAMP-CRP complex then triggers global transcriptional regulation that prepares *E*. *coli* for switching growth from glucose to less favored substrates, such as acetate [34,35].

Interestingly, examining mutations between two pairs of FG and SG isolates from the heterogeneous Z2 and Z10 populations revealed common genetic bases underlying parallel phenotypic diversification (Table 1). Both of the FG isolates, Z2.4 and Z10.2, acquired mutations affecting the mTH gene: Z2.4 had a point mutation in the ribosome binding site of *pntA* (*pntAB* ^2.4^, named after the mutated locus and the evolved isolate) while Z10.2 gained a 4-fold 39 kb amplification encompassing the *pntAB* operon (*pntAB* ^10.2^, S2B Fig.). By contrast, both of the SG isolates Z2.2 and Z10.1 acquired mutations in the EIICB component of PTS (encoded by *ptsG*). Z2.2 gained a point mutation (*ptsG* ^2.2^) that led to a nonsynonymous substitution (G295V). Z10.1 acquired a 10 bp deletion (*ptsG* ^10.1^) that caused frameshift and premature truncation of 415 aa of the 477 aa EIICB protein. Identification of mTH mutations in three populations, particularly the gene amplification by *pntAB* ^10.2^, strongly suggested a growth benefit by increasing mTH expression in the low NADPH-producing *E*. *coli* ZED. The functional importance of *ptsG* ^2.2^, *ptsG* ^10.1^, and other Z-specific mutations, on the other hand, was not self-evident and demanded further investigation.

### Adaptive mutations caused diverse changes in growth rates and diauxic shifts

**Fig 3:**
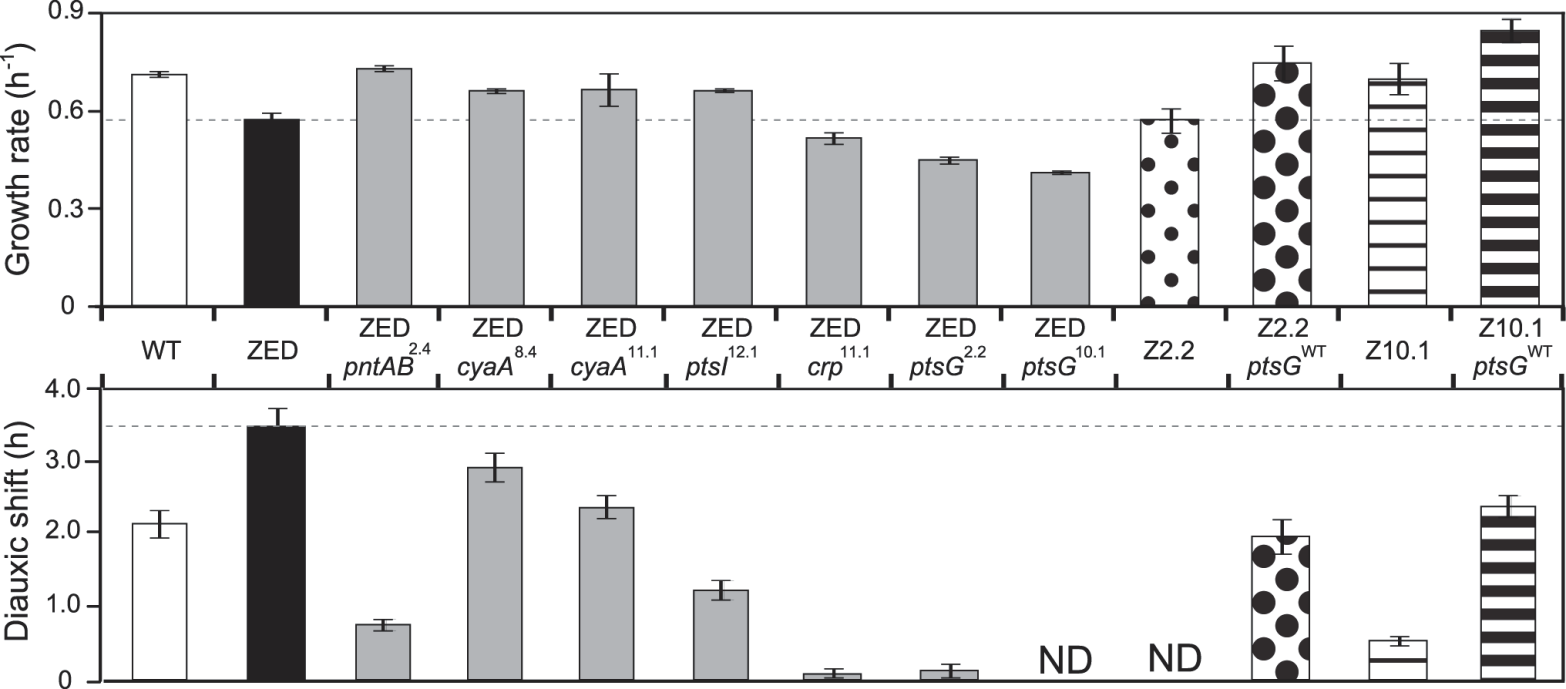
Effects of adaptive mutations on growth rates and diauxic shifts. Dashed lines indicate the phenotype of *E*. *coli* ZED. Error bars are 95% C.I. based on six independent measurements. ND, no diauxic growth.

**Fig 4:**
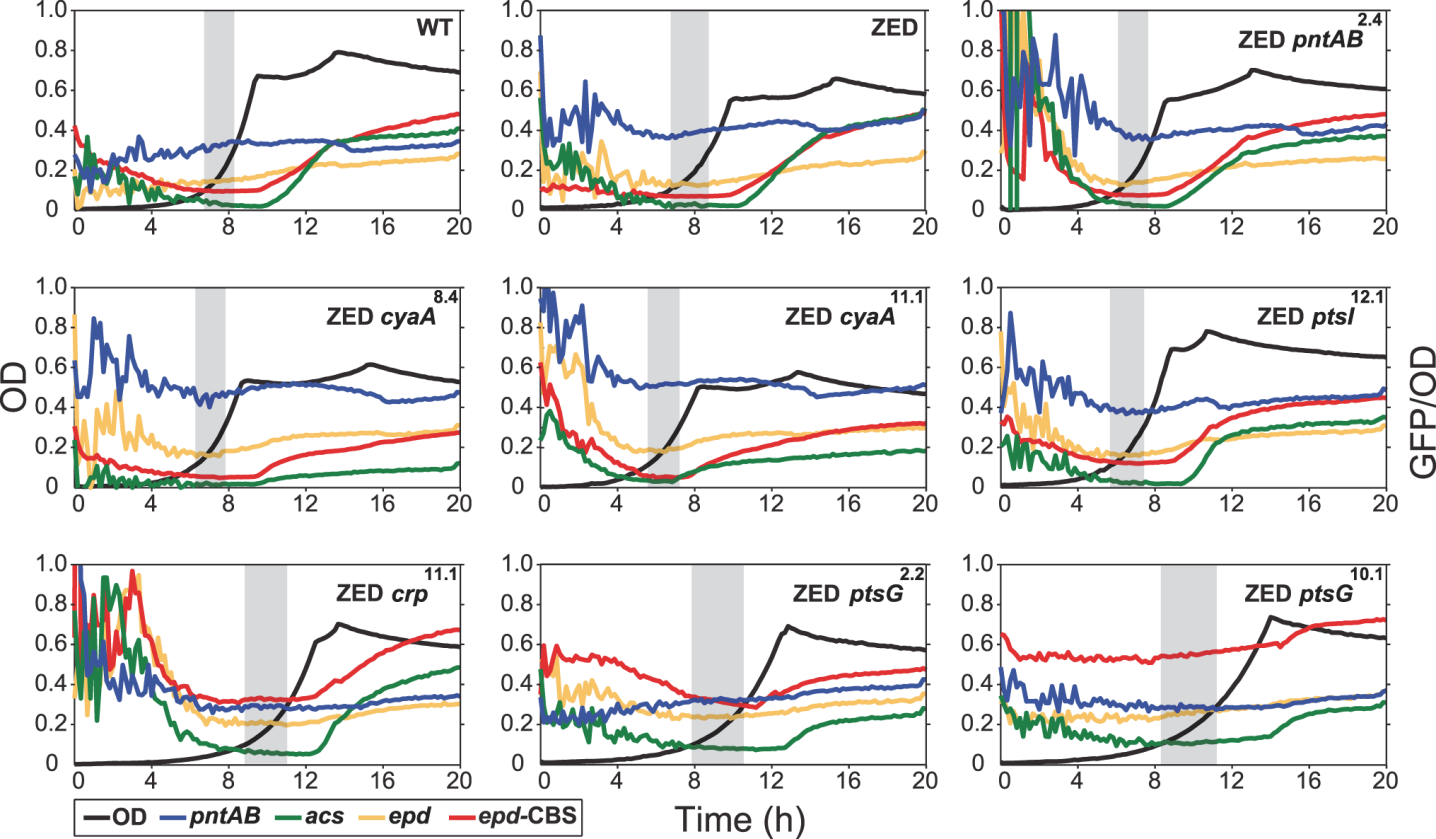
Effects of adaptive mutations on growth profiles and transcriptional activity. Transcriptional activity (defined as GFP/OD) of the *pntAB*, *acs*, and *epd* promoters are scaled by 10^−4^ and the *epd-*CBS hybrid promoter by 10^−5^ to fit into these plots. Transcriptional activity at the exponential phase (defined as OD = 0.1–0.3) is highlighted by a grey window in each panel. Results from one of the three independent experiments are shown as the representative.

To investigate the phenotypic effects of Z-specific mutations, we introduced seven of them into the ancestral ZED background (*pntAB* ^2.4^, *cyaA* ^8.4^, *cyaA* ^11.1^, *crp* ^11.1^, *ptsI* ^12.1^, *ptsG* ^2.2^, *ptsG* ^10.1^). We left out *pntAB* ^12.1^ and two large amplification mutations (*pntAB* ^10.2^, *ptsA* ^10.2^) because the former was a point mutation identical to *pntAB* ^2.4^ and the latter was not amenable to genetic manipulation. These seven mutations caused diverse changes in growth profiles (Fig. 3). Four mutations (*pntAB* ^2.4^, *cyaA* ^8.4^, *cyaA* ^11.1^, *ptsI* ^12.1^) conferred clear selective advantages through increasing growth rates on glucose by 15–27% and shortening diauxic shifts by 16–78%. Among these, *pntAB* ^2.4^ alone was able to restore the growth rate of *E*. *coli* ZED back to the WT level, which indicated the NADPH shortage of the ZED strain as the major cause of its slow growth on glucose. In contrast, the remaining three mutations (*crp* ^11.1^, *ptsG* ^2.2^, *ptsG* ^10.1^) reduced growth rates by 10–28% and nearly or completely abolished diauxic growth (Fig. 3, Fig. 4). Despite the benefit of shortening diauxic shifts, the significant growth rate defect incurred by *crp* and *ptsG* mutations was surprising since they were preserved in lineages thriving through long-term growth selection. Could phenotypes observed here be confounded by epistatic interactions between these and other mutations present in evolved isolates? We tested this possibility by reverting the mutated *ptsG* alleles (*ptsG* ^2.2^, *ptsG* ^10.1^) in two SG isolates Z2.2 and Z10.1 back to wild-type (*ptsG* ^WT^). If *ptsG* ^2.2^ and *ptsG* ^10.1^ exerted an opposite effect in the evolved genetic background, we expected allelic reversion to slow down growth of Z2.2 and Z10.1. Instead, reverting *ptsG* alleles in both evolved isolates increased growth rates by 31% and 21%, respectively (Fig. 3). In addition, allelic reversion lengthened the diauxic shifts of both evolved isolates, consistent with the phenotypes of *ptsG* ^2.2^ and *ptsG* ^10.1^ in the ancestral ZED background. Results indicated that the poor growth of SG isolates on glucose was partly explained by *ptsG* mutations. Moreover, harmful effects of these mutations on glucose growth were qualitatively independent of the genetic context.

### Adaptive mutations exerted distinct influence on the CRP regulation and mTH expression

**Fig 5:**
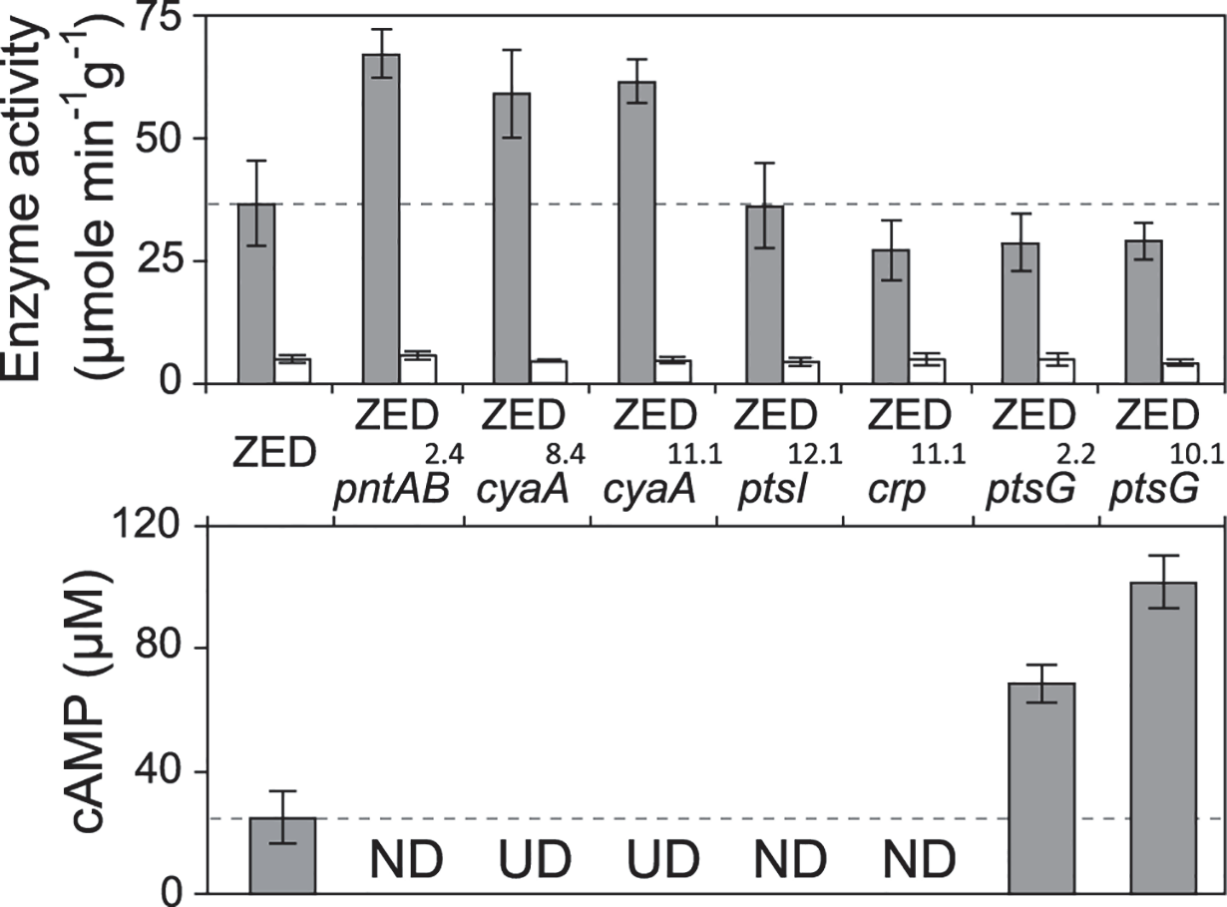
Effects of adaptive mutations on transhydrogenase activity and cAMP concentrations. *In vitro* enzyme activity of mTH and sTH is shown in the upper panel by grey and white bars, respectively. cAMP concentrations are shown in the lower panel. Mutants whose cAMP concentrations are undetectable (UD) or not determined (ND) are indicated. The detection limit of our cAMP quantification is 10 μM. Dashed lines indicate the mTH and cAMP levels of *E*. *coli* ZED. Error bars are 95% C.I. based on three independent measurements.

To unravel the adaptive value of *ptsG* mutations and the physiological bases of Z-specific mutations, we monitored the dynamics of cAMP-CRP regulation and gene expression of *pntAB* throughout the growth cycle using a promoter reporter system based on GFP fluorescence [36]. We focused on cAMP-CRP regulation because CRP is the master regulator of diauxic growth and likely to be affected by mutations in *crp*, *cyaA*, *ptsG*, and *ptsI*. A hybrid promoter consisting of a CRP-binding site (CBS) and the constitutive *epd* promoter was employed to report their influence on the regulatory activity of cAMP-CRP (abbreviated as CRP activity hereafter) [37]. We quantified the transcriptional activity of the *pntAB* promoter as well because prior work suggested that CRP might regulate *pntAB* expression despite the absence of CBS in the *pntAB* operon [34,38]. The dynamics of CRP regulation in *E*. *coli* ZED was similar to WT, characterized by low activity during exponential growth and a steep increase coinciding with the diauxic shift (Fig. 4, Table 2, S3 Table). Between the two strains, disruption of the OPP pathway in *E*. *coli* ZED led to a 15% decrease in CRP activity but a 16% increase in *pntAB* transcription during the exponential phase (operationally defined as OD = 0.1–0.3). The slightly increased *pntAB* transcription in *E*. *coli* ZED was confirmed independently by quantitative PCR (a 2.08 ± 0.38 fold increase relative to WT). Relative to the ZED ancestor, reconstituted mutants ZED *cyaA* ^8.4^ and ZED *cyaA* ^11.1^ (named after the genetic background and allele) showed a 24–49% decrease in CRP activity but a 19–38% increase in *pntAB* transcription. In contrast, ZED *crp* ^11.1^, ZED *ptsG* ^2.2^, and ZED *ptsG* ^10.1^ showed semi-constitutively elevated CRP activity (2.5- to 4-fold) but a 15–24% decrease in *pntAB* transcription. ZED *ptsI* ^12.1^, on the other hand, showed merely 1.4-fold increased CRP activity and no difference in *pntAB* transcription. Quantification of intracellular cAMP of *E*. *coli* ZED and reconstituted mutants bearing *cyaA* and *ptsG* mutations showed a positive correlation between cAMP concentrations and CRP activity (Fig. 4, Fig. 5, Table 2). Above results showed opposite effects of *cyaA* and *ptsG* mutations on cAMP concentrations and CRP activity, leading to divergent regulation of diauxic growth and *pntAB* transcription.

**Table 2:** Effects of adaptive mutations on transcriptional activity during the exponential phase.

Genotype	Transcriptional activity (GFP/OD)^a^				
	*pntAB*	*acs*	*epd*-CBS	*epd*	CRP^b^
WT	3284 ± 150	299 ± 68	9555 ± 51	1479 ± 43	6.5 ± 0.2
ZED	3802 ± 104	263 ± 42	6996 ± 9	1283 ± 61	5.5 ± 0.3
ZED *pntAB* ^2.4^	3670 ± 95	263 ± 59	7518 ± 49	1370 ± 80	5.5 ± 0.4
ZED *cyaA* ^8.4^	4476 ± 183	166 ± 16	5318 ± 306	1647 ± 33	3.2 ± 0.2
ZED *cyaA* ^11.1^	5137 ± 76	320 ± 5.1	5300 ± 142	1874 ± 70	2.8 ± 0.2
ZED *crp* ^11.1^	2937 ± 52	620 ± 31	32992 ± 205	2103 ± 8.5	15.7 ± 0.1
ZED *ptsG* ^2.2^	3221 ± 61	850 ± 43	32364 ± 932	2389 ± 28	13.6 ± 0.5
ZED *ptsG* ^10.1^	2886 ± 20	1095 ± 41	54634 ± 389	2547 ± 60	21.5 ± 0.4
ZED *ptsI* ^12.1^	3836 ± 44	294 ± 64	12581 ± 292	1657 ± 29	7.6 ± 0.0

^a^ Measurements are averaged across the range of OD = 0.1–0.3. Data are reported as means and 95% confidence intervals of averaged measurements from three replicate experiments.

^b^CRP activity is defined as the transcription driven by the *epd*-CBS hybrid promoter divided by that of the constitutive *epd* promoter.

**Fig 6:**
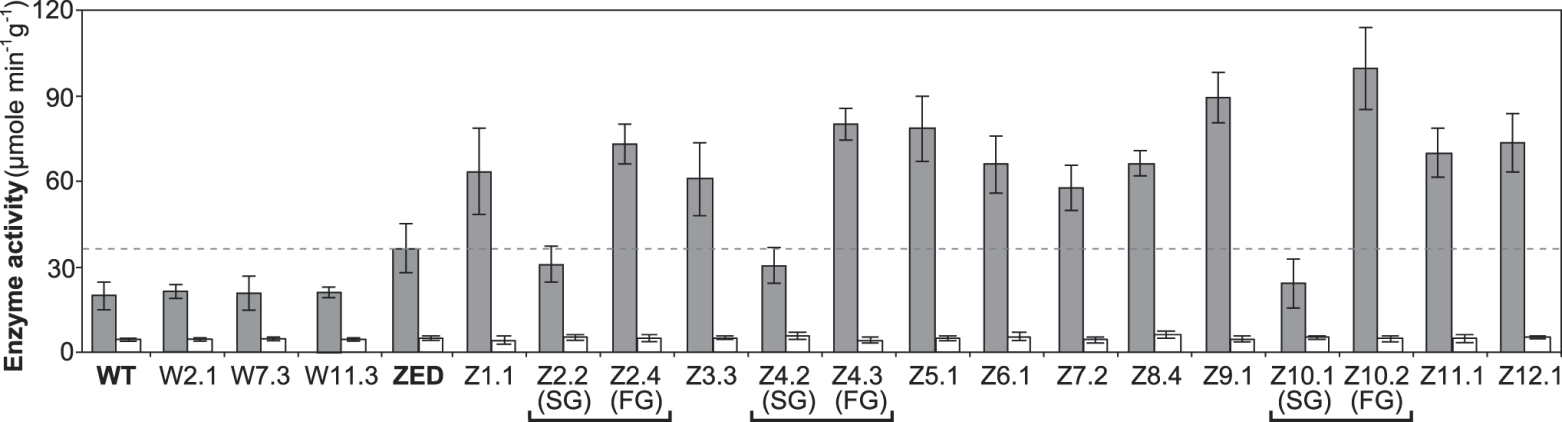
Transhydrogenase activity of *E*. *coli* WT, *E*. *coli* ZED, and evolved isolates. *In vitro* enzyme activity of mTH and sTH is shown by grey and white bars, respectively. The dashed line indicates the mTH activity of *E*. *coli* ZED. Error bars are 95% C.I. based on three independent measurements. Evolved isolates from the same population are grouped by a square bracket. Slow-growing and fast-growing isolates are indicated by SG and FG, respectively.

Did changes in the *pntAB* transcription reflect in the mTH enzyme level and alter the redox cofactor concentrations? We quantified the mTH activity of *E*. *coli* WT, *E*. *coli* ZED and the seven reconstituted mutants (Fig. 5, Fig. 6). As a control we also measured the enzyme activity of sTH, which catalyzed the reverse hydride transfer reaction. While the sTH activity was similar across characterized strains, the mTH activity differed significantly. Relative to WT, *E*. *coli* ZED showed a nearly 2-fold increase in the mTH activity. In the ZED background, the mTH activity was increased further by 1.6- to 1.8-fold by *pntAB* ^2.4^, *cyaA* ^8.4^, and *cyaA* ^11.1^, not affected by *ptsI* ^12.1^, and slightly decreased by *crp* ^11.1^, *ptsG* ^2.2^, and *ptsG* ^10.1^ (*P* > 0.05). The influence of *pntAB* ^2.4^, a *cis* mutation in the ribosome binding site, could be explained by directly enhancing mTH protein translation. By contrast, *cyaA* ^8.4^, *cyaA* ^11.1^, *crp* ^11.1^, *ptsG* ^2.2^, and *ptsG* ^10.1^ likely affected mTH expression through alleviating or aggravating the CRP-imposed transcriptional repression of *pntAB* (Fig. 4, Table 2). To see if mTH activity affected the redox cofactor concentrations, we quantified NAD(H) and NADP(H) in *E*. *coli* ZED, two mutants exhibiting higher mTH expression (ZED *cyaA* ^8.4^, ZED *cyaA* ^11.1^), and two mutants with slightly lower mTH expression (ZED *ptsG* ^2.2^, ZED *ptsG* ^10.1^). LC-MS quantification of these strains indicated their cofactor concentrations indistinguishable from *E*. *coli* WT (S4 Table) [14]. This result was consistent with an earlier conclusion that the production and consumption rates of redox cofactors were tightly coupled in metabolism [16,19]. As such, reducing NADPH production in *E*. *coli* ZED also slowed down its anabolic consumption and cellular growth, which collectively led to comparable steady-state cofactor concentrations.

Functional characterization of adaptive mutations revealed their distinct influence on CRP activity and confirmed the growth benefit of increased mTH expression by *cis* or *trans* regulation. Moreover, diverse effects of these mutations on growth rates and diauxic shifts re-echoed the remarkable phenotypic variation among the Z populations.

### mTH contributed to the robustness and parallel evolution of *E*. *coli* ZED

Upregulation of mTH in *E*. *coli* ZED suggested that this enzyme actively buffered the NADPH perturbation due to losing the OPP pathway (Fig. 6). Is mTH upregulation essential to maintain the physiological robustness of the ZED strain? We disrupted the *pntAB* operon of *E*. *coli* WT and ZED and studied their growth phenotypes in M9 glucose medium or in the nutrient-rich LB medium where metabolism was less constrained (Table 3). Deletion of *pntAB* did not affect the growth rate of WT under either condition. By contrast, while *pntAB* deletion marginally affected *E*. *coli* ZED in LB medium, it reduced the growth rate by 90% in M9 glucose medium. The harmlessness of *pntAB* deletion to *E*. *coli* WT suggested that mTH, unlike the OPP pathway, was not the primary contributor to NADPH production. Given its significance in maintaining the physiological robustness of *E*. *coli* ZED, mTH may function as flexible backup in metabolic networks to soothe cofactor perturbations resulting from changes in the pathway structure or growth conditions. Interestingly, deletion of *pntAB* caused a prolonged diauxic shift of WT in M9 glucose medium, suggesting an unidentified role of mTH in controlling diauxic growth.

**Table 3:** Phenotypes of Δ*pntAB*.

	WT	ZED
**Growth rate in LB (h^-1^)**		
No deletion	1.71 ± 0.12	1.41 ± 0.06
Δ*pntAB*	1.75 ± 0.14	1.24 ± 0.15
**Growth rate in M9 glucose (h^-1^)**		
No deletion	0.66 ± 0.01	0.56 ± 0.01
Δ*pntAB*	0.64 ± 0.03	0.06 ± 0.02
**Diauxic shift in M9 glucose (h)**		
No deletion	2.10 ± 0.21	3.47 ± 0.24
Δ*pntAB*	2.91 ± 0.14	ND^a^

Data are reported as means ± 95% C.I. based on five independent measurements.

^a^No diauxic growth.

Above results showed mTH as a buffer at the initial stage of pathway evolution. Does mTH remain crucial in the long run? Genome sequencing and functional characterization identified mTH-upregulating mutations emerged in five Z populations (Table 1, Fig. 5). To check the prevalence of mTH in adaptive evolution, we quantified the mTH activity of one FG and one SG isolates from each of the three heterogeneous populations (Z2, Z4, Z10), one isolate from each of the nine homogenous populations (Z1, Z3, Z5-9, Z11-12), and three sequenced W isolates (W2.1, W7.3, W11.3) that showed different levels of paraquat tolerance (Fig. 2). While the three W isolates showed mTH activity comparable to their WT ancestor, we observed 1.6–2.8 fold increased mTH activity in Z isolates from the nine homogenous populations and in FG isolates (Z2.4, Z4.3, Z10.2) from the remaining three heterogeneous populations relative to their ZED ancestor (*P* < 0.05, Fig. 6). By contrast, the three SG isolates (Z2.2, Z4.2, Z10.1) showed 15–32% decreases in mTH activity (*P* > 0.05). As a control experiment we also quantified the sTH activity and found it indistinguishable across examined isolates.

Functional characterization of mTH in *E*. *coli* ZED and Z evolved isolates suggested this broadly distributed enzyme (S3 Fig.) as a prominent player in redox cofactor homeostasis on both physiological and evolutionary timescales. Yet the causes of phenotypic diversification in heterogeneous Z populations, particularly the adaptive values of *ptsG* mutations in SG isolates, remained to be elucidated.

### Adaptive diversification to restore the NADPH production through glucose/acetate co-utilization

The semi-constitutively elevated CRP activity in reconstituted mutants ZED *ptsG* ^2.2^ and ZED *ptsG* ^10.1^ offered clues about the growth advantage of *ptsG* mutations besides shortening diauxic shifts (Fig. 3, Fig. 4). This CRP phenotype resembles the glucose starvation responses in *E*. *coli* PTS knockouts where disruption of PTS decelerates glucose transport, reduces acetate secretion, and enables *E*. *coli* to co-utilize unfavorable substrates due to the relief of catabolite repression (i.e. glucose preference) by cAMP-CRP [39–42]. Could *ptsG* ^2.2^ and *ptsG* ^10.1^ from SG isolates act similarly by allowing co-utilization of glucose and acetate, the latter of which is less favored but excreted abundantly during glucose batch culture? If so, acetate consumption through isocitrate dehydrogenase of the TCA cycle would provide a unique physiological benefit to *E*. *coli* ZED by generating extra NADPH to complement the NADPH shortage solely through glucose metabolism [19] (Fig. 1).

**Fig 7:**
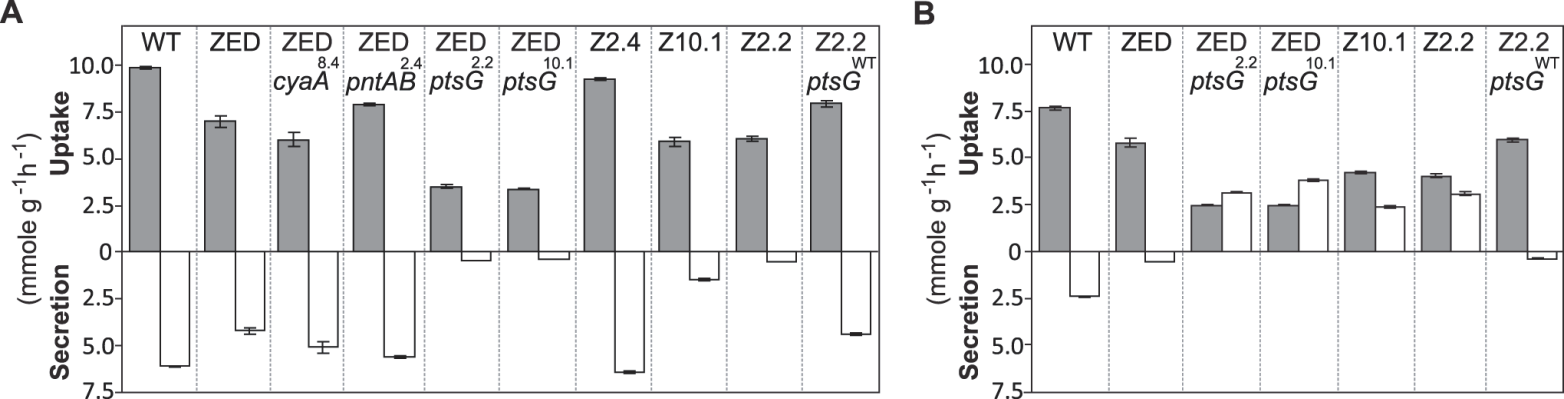
Substrate uptake and secretion rates. Uptake and secretion rates of glucose (grey) and acetate (white) were determined during growth of *E*. *coli* in M9 minimal medium supplemented with either glucose (3 g/l) (A) or glucose (2 g/l) plus sodium acetate (2 g/l) (B). Error bars are 95% C.I. based on three independent measurements.

We first investigated if *ptsG* ^2.2^ and *ptsG* ^10.1^ allowed glucose/acetate co-utilization of *E*. *coli* ZED by examining their influence on the expression of a key gene (*acs*, encoding acetyl-CoA synthetase) for acetate metabolism and on the substrate uptake and secretion profile in M9 glucose medium plus various concentrations of acetate. In *E*. *coli* WT and ZED, *ac*s expression was kept low during exponential growth on glucose and upregulated by cAMP-CRP during the diauxic shift (Fig. 4) [35]. Nevertheless, in ZED *ptsG* ^2.2^ and ZED *ptsG* ^10.1^ the elevated CRP activity resulted in the semi-constitutive *acs* expression, suggesting the physiological competence to utilize acetate throughout the growth cycle. Corroborating this finding, ZED *ptsG* ^2.2^, ZED *ptsG* ^10.1^, and SG isolates Z2.2 and Z10.1 exhibited minimal acetate secretion during growth on glucose and consumed glucose and acetate simultaneously when both substrates were present (Fig. 7, S4 Fig.). The effect of *ptsG* mutations on co-utilization was further confirmed by the loss of this phenotype in Z2.2 when reverting its *ptsG* ^2.2^ allele back to *ptsG* ^WT^. By contrast, reconstituted mutants ZED *pntAB* ^2.4^ and ZED *cyaA* ^8.4^, and particularly the FG isolate Z2.4 showed significantly increased acetate secretion, consistent with a cross-feeding scenario where the SG isolates utilized acetate secreted by FG isolates in the same population to fuel NADPH production.

**Fig 8:**
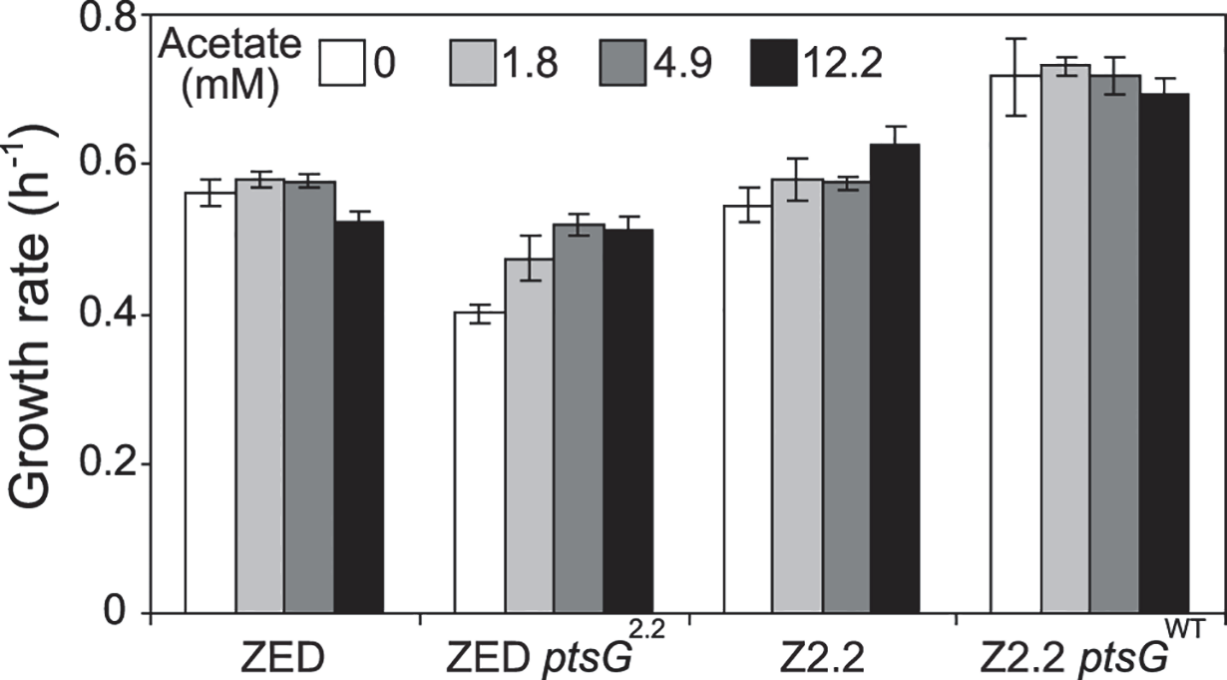
Growth rates of *E*. *coli* in response to acetate concentrations. Strains were grown in M9 glucose (1 g/l) medium supplemented with various amounts of acetate. Error bars are 95% C.I. based on four independent measurements.

**Fig 9:**
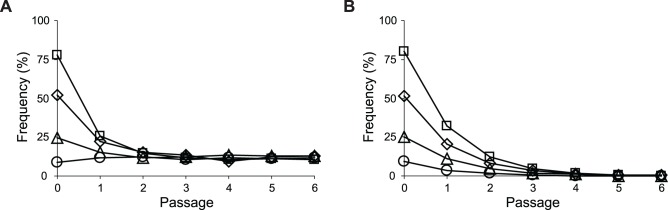
Frequencies of *E*. *coli* during growth competition. SG isolate Z2.2 (A) or its *ptsG* revertant Z2.2 *ptsG* ^WT^ (B) were mixed with a FG isolate Z2.4 at various starting ratios and co-cultured in M9 glucose (1 g/l) medium over six passages. Two replicate cultures were performed for each starting ratio. Results from one replicate culture are shown due to good reproducibility.

Does the glucose/acetate co-utilization conferred by *ptsG* ^2.2^ and *ptsG* ^10.1^ improve growth of *E*. *coli* ZED? We characterized growth of *E*. *coli* ZED and the SG isolate Z2.2 with either *ptsG* ^WT^ or *ptsG* ^2.2^ alleles in M9 glucose medium supplemented with various concentrations of acetate (Fig. 8, S5 Fig.). While *ptsG* ^2.2^ increased growth rates in response to increasing concentrations of acetate under both genetic contexts, the phenotypic effect of *ptsG* ^WT^ was context-dependence. *ptsG* ^WT^ decelerated growth of the ZED strain but not Z2.2 under high acetate concentrations. Are growth benefits conferred by co-utilization and shortening diauxic shifts sufficient to compensate the cost of *ptsG* mutations on glucose growth and allow SG isolates to compete with FG isolates from the same population? We demonstrated the adaptive values of *ptsG* mutations by monitoring the growth competition between Z2.2 and Z2.4 or between Z2.2 *ptsG* ^WT^ and Z2.4 with different starting ratios in M9 glucose medium. Despite a 30% increase in the glucose growth rate through the allelic reversion (Fig. 8), Z2.2 *ptsG* ^WT^ was outcompeted by Z2.4 at all starting ratios tested within 6 growth passages (Fig. 9). On the contrary, Z2.2 was able to co-exist with Z2.4 from all starting ratios and converged to a level (10–13%) similar to the allelic frequency of *ptsG* ^2.2^ in the end-point Z2 population (16.9 ± 4.7%) estimated by quantitative PCR.

## Discussion

Through genetic and physiological dissection of evolved isolates, we showed enhancing mTH expression was the predominant evolutionary change to buffer the NADPH perturbation across twelve replicate populations founded by *E*. *coli* ZED. This recurrence seems surprising given the existence of alternative solutions in metabolism, such as flux rerouting, expression of isoenzymes, or converting the cofactor specificity of enzymes [14–18]. Below we suggest potential functional constraints and methodological caveats that might have prevent their emergence or discovery in our study. First, the implementation of alternative NADPH-generating strategies may require more than one mutational step. If any single mutation is insufficient to provide a growth benefit, mutations required to establish these strategies will rarely be assembled under constant selective pressure imposed by laboratory evolution. For instance, theoretically it should be possible to reroute metabolic flux through the NADP-dependent malic enzyme (MaeB) for NADPH production [43] (Fig. 1). Yet this implementation might require three mutational steps: (1) upregulating the MaeB expression, (2) increasing the production of its substrate malate, and (3) preventing the accumulation of its product pyruvate. Similarly, although protein engineering and studies of enzyme homologues have demonstrated the possibility to convert NAD-dependent enzymes, like the NAD kinase, malate dehydrogenase, glyceraldehyde-3-phosphate dehydrogenase, and lipoamide dehydrogenase, for NADPH production, these often require multiple mutations and have to go through function-inferior intermediates [44–47]. Second, our knowledge of *E*. *coli* ZED genome evolution was limited by sampling just seven evolved isolates, even though these phenotypically diverse isolates were selected with the intention to capture the genetic diversity underlying the high phenotypic variation among Z populations. It is possible that evolved isolates acquiring alternative NADPH-producing strategies are present at low frequencies in the population, just like the SG isolates with *ptsG* mutations (Fig. 9). Third, we characterized only evolved isolates from the end-point populations. Lineages existing early on might be outcompeted because their NADPH-generating strategies are more pleiotropic and not as competitive or evolvable as those harboring the mTH-upregulating mutations [48]. Future work employing community sequencing of the Z populations over time may unravel other NADPH-producing strategies, and may explain why they become extinct during evolution.

Intriguingly, the evolutionary significance of mTH is corroborated by laboratory evolution of a highly divergent species *Methylobacterium extorquens* [49]. An engineered strain of *M*. *extorquens* bearing a NADPH-underproducing pathway was evolved to improve its growth on the single-carbon compound methanol. Not only did the engineered ancestor immediately upregulate mTH expression to buffer the cofactor imbalance, but long-term evolution also led to further elevation of the mTH activity in all eight replicate populations. Given that 2500 million years of divergence between *E*. *coli* and *M*. *extorquens* has led to substantial differentiation in their genomes (4.6 vs. 6.9 Mb), metabolism (multi- vs. single-carbon assimilation), and ecology (animal- vs. plant-association) [22,50,51], the genetic parallelism underlying their convergent adaptation to NADPH perturbations is unlikely to be explained by chance. Rather, this remarkable similarity suggests the influence of genetic architecture on constraining evolutionary trajectories [52,53] and underscores the functional importance of mTH beyond its currently appreciated role in physiological robustness [19,54]. mTH, distributed broadly across the three domains of life (S3 Fig.), may promote the evolvability of metabolic networks in two ways. First, it flattens the genotype-phenotype landscape [55] through ameliorating catastrophic hub perturbations accompanied by pathway modifications. This phenotypic robustness allows organisms to traverse fitness-inferior states (so-called fitness valleys), similar to the effect of a robust protein fold to tolerate function-innovating but structure-destabilizing mutations [56,57], the flexibility of gene networks to adopt new regulation without compromising pre-existing functions [58,59], or the Hsp90 chaperone to promote the accumulation of cryptic genetic variation in morphological evolution [60]. Following this transition, the ability of mTH to modulate the NAD(P)H pools through a single reaction offers metabolism a quick and less pleiotropic solution to regain the redox balance, compared to the requirement of accumulating multiple mutations in order to reroute metabolic flux or switch the cofactor specificity of enzymes. The versatility of mTH in adaptation is also reflected by *E*. *coli* Δ*pgi* experiencing the opposite physiological challenge [12,19]. During growth on glucose, the blocked glycolysis in *E*. *coli* Δ*pgi* caused overproduction of NADPH through the OPP pathway and induced downregulation of the mTH expression to counteract such disturbance. Laboratory evolution of *E*. *coli* Δ*pgi* led to the acquisition of mTH-attenuating mutations in four of the ten populations to restore the NADPH homeostasis.

In addition to the prevalent mTH upregulation, probing the cause of high phenotypic variation among Z isolates revealed a minor NADPH-replenishing strategy relying on glucose/acetate co-utilization. *E*. *coli* typically prefers glucose over acetate as the growth substrate, and the expression of the acetate-assimilating gene *acs* is repressed whenever glucose is present [35]. However, *ptsG* mutations in SG isolates altered such substrate preference by decelerating glucose transport and enabling simultaneous acetate uptake through the semi-constitutive *acs* expression (Fig. 4, Fig. 7B). Through cross-feeding on acetate excreted by FG isolates in the same population, SG isolates were able to gain a growth advantage by producing extra NADPH from the TCA cycle (Fig. 8, S5 Fig.). A similar co-utilization phenotype has been discovered in *E*. *coli* WT evolved in glucose-limited chemostat cultures [61], but the underlying mutations and physiological effects differ. In this case, promoter mutations of the *acs* gene enhanced acetate uptake without compromising glucose transport in order to scavenge any available growth substrate in a nutrient-scarce environment. Moreover, unlike these *acs* mutations, *ptsG* mutations in SG isolates not only permitted substrate co-utilization but greatly shortened the diauxic shift (Fig. 3). Combining these two advantages, SG isolates were able to persist with FG isolates at low frequencies in the population despite suffering slower glucose transport (Fig. 7A, Fig. 9A). This coexistence of SG and FG isolates bears resemblance to the ecological differentiation of *E*. *coli* WT evolved in batch cultures supplemented with glucose and acetate [62]. Instead of selecting for a co-utilization generalist, evolution under this dual-substrate condition gave rise to two coexisting ecotypes, both retaining the preference of glucose over acetate. The “fast switcher” grew slowly on glucose but switches quickly to acetate upon glucose depletion. By contrast, the “slow switcher” grew faster on glucose but suffers a longer diauxic shift.

Aside from accelerating growth on glucose, the intense selection to shorten the prolonged diauxic shift of *E*. *coli* ZED has been reflected in the phenotypic effect of all Z-specific mutations (Fig. 3). Among these, we were particularly interested in *pntAB* mutations as this mTH-encoding gene has not been implicated in the control of diauxic growth [35]. The involvement of *pntAB* in diauxic growth was also supported by a 40% extended diauxic shift of *E*. *coli* WT due to the *pntAB* deletion (Table 3). Notably, the influence of *pntAB* on diauxic growth appeared independent of the canonical cAMP-CRP regulation, since both the CRP activity and *acs* expression were indistinguishable between *E*. *coli* ZED and reconstituted mutant ZED *pntAB* ^2.4^ (Fig. 4). What could the function of mTH be? Even though mTH is not required for NADPH production in the ensuing acetate growth phase [19], its ability to directly modulate the NAD(P)H pools independent of catabolizing growth substrates might support the energy demand from changing the expression of hundreds of genes during the diauxic shift [63]. Dynamic transcriptomic and metabolomic profiling of *E*. *coli* WT and *E*. *coli* Δ*pntAB* during this physiological transition should clarify the exact mechanism and validate this assumption.

Our study unravels the significance of a conserved buffering mechanism in metabolic evolution. Results suggest that mechanisms dedicated to mitigating hub perturbations may promote not only the robustness but also evolvability of metabolic networks. It would be interesting to test the generality of this finding by genetically perturbing other prominent hub metabolites, like ATP and glutamine, and examining if corresponding conserved buffering mechanisms (i.e. phosphofructokinase and the nitrogen regulatory protein GlnB, respectively) would play a critical role as mTH during adaptation [31]. Moreover, comparing adaptation of *E*. *coli* WT and ZED shows how slight changes in the pathway structure could lead to distinct evolutionary outcomes, as demonstrated by the genotypic and phenotypic differentiation between W and Z isolates (Table 1, Fig. 2). Interestingly, despite the removal of the OPP pathway, several Z isolates evolved growth rates as high as the W isolates in about a thousand generations. Dissecting the metabolic flux distribution in these two lineages and the contribution of individual mutations will provide a mechanistic understanding of the evolution of flux phenotypes. Such knowledge will be valuable for engineering redox cofactor production to sustain the biosynthesis of valuable compounds [64]. Furthermore, we anticipate that experimental evolution combined with network analysis will be able to elucidate more conserved features of biological systems that promote the robustness, evolvability and convergent evolution in different evolutionary lineages.

## Materials and Methods

### Growth media

All chemicals were purchased from Sigma-Alderich or Fisher Scientific. One liter of Luria-Bertani (LB) medium consists of 10 g of tryptone, 5 g of yeast extract, and 10 g of NaCl in one liter of deionized water. One liter of M9 minimal medium consisted of 5.98 g of Na_2_HPO_4_, 3 g of KH_2_PO_4_, 0.5 g of NaCl, 0.8 g of NH_4_Cl, 976.7 ml of deionized water, and the following components that were filter-sterilized separately and then added immediately before use: 1 ml of 0.1 M CaCl_2_, 2 ml of 1 M MgSO_4_, 0.2 ml of 185 mM FeCl_3_, 0.3 ml of 1 mM thiamine hydrochloride, 9.8 ml of M9 trace element solution, and 10 ml of carbon sources. M9 trace element solution consisted of 0.18 g of ZnSO_4_·7H_2_O, 0.12 g of CuCl_2_·2H_2_O, 0.12 g of MnSO_4_·H_2_O, 0.18 g of CoCl_2_·6H_2_O in 980 ml of deionized water. The carbon sources were either glucose or sodium acetate dissolved in deionized water. Solid medium was made by supplementing one liter of liquid medium with 20 g of agar.

### Plasmid and strain construction

Plasmids used in this study are listed in S5 Table. All enzymes used for plasmid construction were purchased from New England Biolabs. Unmarked allelic exchange plasmids for deleting genes or introducing adaptive mutations were constructed based on pHC140 and maintained in *E*. *coli* PIR1 (Life Technologies). This *sacB*-based suicide plasmid was generated by digestion of pDS132 [65] with *Sbf*I and *Sac*I followed by ligation of the 5.2-kb fragment with a 42-bp polylinker formed by annealing oligonucleotides linker.F and linker.R (S6 Table). Plasmids designed to delete the *pntAB* operon contain a synthetic Δ*pntAB* allele generated through PCR splicing [66]. Upstream and downstream regions of *pntAB* were PCR amplified by primer pairs HCEp64A/HCEp65 and HCEp66/HCEp67, respectively. The Δ*pntAB* allele was created by overlapping extension of the upstream and downstream fragments followed by ligation with *Nhe*I/*Xho*I-digested pHC140 to generate pHC145. Plasmids designed to introduce *cyaA* ^8.4^, *ptsG* ^WT^ (with respect to *ptsG* ^10.1^), *ptsG* ^10.1^, *ptsI* ^12.1^, *ptsG* ^WT^ (with respect to *ptsG* ^2.2^), *ptsG* ^2.2^, *pntAB* ^2.4^, *cyaA* ^11.1^, and *crp* ^11.1^ alleles were constructed in a similar manner. Nine 1.2-kb PCR fragments containing each of these alleles were amplified by primer pairs HCEp111/HCEp112, HCEp115/HCEp116, HCEp115/HCEp116, HCEp119/HCEp120, HCEp123/HCEp124, HCEp123/HCEp124, HCEp127/HCEp128, HCEp131/HCEp132, and HCEp135/HCEp136, followed by ligation with *Nhe*I/*Xho*I-digested pHC140 to generate pHC150e, pHC151w, pHC151e, pHC152e, pHC153w, pHC153e, pHC154e, pHC155e, and pHC156e, respectively. Plasmid pHC179 for creating fluorescently labeled *E*. *coli* was constructed in four steps. From a former construct pHC08 [67] a gene cassette consisting of *P*_*tacA*_ *-mCherry* surrounded by transcription terminators *t*_*rrnB*_ and *t*_*T7*_ was PCR amplified by primer pair HC161p1/HC161p2 and ligated with *Sph*I/*Spe*I-digested pHC140 to generate pHC161m. The downstream region of the *araBAD* operon was PCR amplified by primer pair HCEp161/HCEp162 and ligated with *PspOM*I/*Spe*I-digested pHC161m to generate pHC175. The upstream region of the *araBAD* operon was PCR amplified by primer pair HCEp163/HCEp164 and ligated with *Nhe*I/*Sac*I-digested pHC175 to generate pHC176. Finally the *P*_*tacA*_ promoter of pHC176 was removed by *Mlu*I/*Bsa*I double digestion followed by ligation with the bacteriophage promoter *P*_*A1*_ [68] formed through annealing oligonucleotides PA1.F and PA1.R to generate pHC179.

*E*. *coli* bearing gene deletions or adaptive mutations was generated by an established method [65]. Allelic exchange plasmids were introduced into *E*. *coli* through electroporation. Isolates with plasmids integrated into the chromosome were selected on LB agar supplemented with chloramphenicol (25 mg/l). These isolates were then spread on LB agar with 5% sucrose and without NaCl to select for loss of the *sacB* gene through plasmid excision. The genotypes of resultant mutants were confirmed by colony PCR with allele-specific primers listed in S6 Table. Cells were suspended in phosphate buffered saline (PBS) with 7.5% dimethyl sulfoxide (DMSO, v/v) and preserved at −80°C.

### Evolution experiments

The W and Z populations, each consisting of 12 replicates, were founded by *E*. *coli* MG1655 WT (obtained from Deutsche Sammlung von Mikroorganismen und Zellkulturen GmbH) and *E*. *coli* MG1655 ZED [19], respectively. All populations were grown in 640 μl of M9 medium supplemented with glucose (1 g/l) contained in 48-well microtiter plates (Corning) and incubated in a 37°C shaking incubator at 300 rpm. Over the 113 passages 1.25 μl of the stationary-phase cultures was transferred daily into fresh growth medium (corresponding to 512-fold dilution and an average of 9 cell generations per passage). This transfer protocol ensured that all populations completed growth prior to the next daily transfer. The population size thus fluctuated between 2 × 10^6^ and 10^9^. Samples of evolved populations were collected every two weeks, supplemented with 7.5% DMSO (v/v), and preserved at −80°C for later analysis. From each of the end-point replicate populations, four evolved isolates were selected based on their colony morphology formed on M9 glucose (5 g/l) agar for further characterization (S1 Table).

### Genome resequencing and locus sequencing

Genomic DNA was extracted by DNeasy Blood & Tissue Kit (QIAGEN) following the protocol for Gram-negative bacteria. Construction of tagged paired-end genomic libraries and sequencing were performed by GATC Biotech (Konstanz, Germany). Paired-end genomic libraries were sequenced by the Illumina HiSeq 2000 platform. Paired-end reads, each of 100 bp, were aligned to the reference genome of *E*. *coli* MG1655 (GenBank accession no. U00096.3) [23] by CLC Genomics Workbench (CLC bio) to identify mutations. The identity of each mutation was validated by manually checking the read alignments.

The sequence of *pntAB*, *cyaA*, and *pykF* loci was confirmed by Sanger sequencing. Two primer pairs, HCEp177/HCEp178 and HCEp179/HCEp180, were used to amplify and sequence two fragments (1.8 kb and 1.6 kb, respectively), which together spanned the entire *pntAB* operon plus its 150 bp upstream and 60 bp downstream regions. Two primer pairs, HCEp181/HCEp182 and HCEp183/HCEp184, were used to amplify and sequence two fragments (1.8 kb and 0.9 kb, respectively), which collectively covered the entire *cyaA* gene and its 50 bp upstream region. One primer pair HCEp185/HCEp186 was used to amplify and sequence a 1.8 kb fragment encompassing the entire *pykF* gene plus its 50 bp upstream and 150 bp downstream regions. Each fragment was amplified by colony PCR and purified by QIAquick PCR Purification Kit (QIAGEN). Sanger sequencing of purified fragments was performed by Eurofins Genomics (Ebersberg, Germany).

### Growth profiling

Each growth experiment began with the inoculation of 1 μl of frozen stocks into 200 μl LB medium contained in 96-well flat microtiter plates (Nunc) and incubated overnight in a 37°C shaking incubator at 500 rpm. From the LB precultures 1 μl was transferred to 200 μl M9 glucose (1 g/l) medium and incubated under identical conditions. Subsequently, 1 μl of M9 precultures was transferred to 200 μl M9 medium supplemented with desired carbon sources. For each strain, optical densities (OD) at 600 nm of 3–6 replicate cultures incubated at 37°C with constant shaking were monitored using a TECAN infinite M200 plate reader at 10 min intervals. Growth profiles of *E*. *coli* incubated in this plate reader were consistent with those through shake flask cultivation [69]. OD readouts from this plate reader were multiplied by a factor of 2.2 to make them comparable to those reported by a typical spectrophotometer with 1 cm path length. Growth rates, yields (as maximum OD), and diauxic shifts were determined by Curve Fitter [70]. Growth rates at the exponential phase were computed as the slope of the regression line of the natural logarithm of OD against the incubation time in the range of OD 0.05–0.35. To detect diauxic growth, a second slope value was computed by extending the OD range of linear regression to include the later growth phase (i.e. from 0.05 to 90% maximum OD of each isolate). Relative to the slope computed by linear regression of OD 0.05–0.35, the presence of diauxic growth at the later growth phase would significantly lower the second slope value. By contrast, the two slope values were statistically indistinguishable (i.e. *P* > 0.05) for isolates exhibiting just a single growth phase on glucose.

### Enzyme assays

The activity of transhydrogenases was quantified by an established method [14]. Exponentially growing cells at an OD between 0.45 and 0.6 in M9 glucose (3 g/l) medium were harvested by centrifugation at 4°C and washed twice with chilled PBS. Cells were suspended in a cell lysis buffer (100 mM Tris-HCl, pH 7.5, 5 mM MgCl_2_, 1 mM dithiothreitol, 0.16 mM phenylmethylsulfonyl fluoride) and disrupted by French press. Cell debris was removed from cell extracts by centrifugation at 23000 g for 30 min at 4°C. The membrane fraction and the membrane-free soluble fraction were further separated by centrifugation at 159000 g and 4°C for 3 h. The membrane fraction was resuspended in the cell lysis buffer. Protein concentrations of both fractions were quantified by the Bradford method [71]. mTH activity in the membrane fraction and sTH activity in the soluble fraction were assayed as three replicates at 30°C in 200 μl of the cell lysis buffer supplemented with 0.5 mM NADPH and 1 mM 3-acetylpyridine adenine dinucleotide (APAD^+^). Changes in absorbance at 400 nm and 310 nm due to the reduction of APAD^+^ and the oxidation of NADPH, respectively, were monitored simultaneously by a TECAN infinite M200 plate reader at 1 min intervals.

### Quantitative PCR

To quantify gene expression, exponentially growing cells at an OD between 0.45 and 0.6 in M9 glucose (3 g/l) medium were harvested by adding 1/10^th^ the volume of a growth-stopping solution (5% Tris-EDTA saturated phenol and 95% ethanol) followed by centrifugation at 9000 g for 5 min at 4°C. Total RNA was extracted using the RNeasy Mini Kit (QIAGEN), followed by removal of residual genomic DNA with the Turbo DNA-free Kit (Ambion). cDNA for real-time PCR was synthesized by the GoScrip Reverse Transcription System (Promega). The primer pairs used to amplify and detect transcripts of *pntAB* and *rpoD* genes were HCEp19/HCEp20 and HCEp15/HCEp16, respectively (S6 Table). Real-time PCR was performed in three replicates with the SsoAdvanced SYBR Green Supermix (Bio-Rad) on a CFX Connect Real-Time PCR System (Bio-Rad) according to the manufacturer’s instructions. The *rpoD* gene (encoding the sigma 70 factor of the RNA polymerase) was chosen as the reference for data normalization. Changes in gene expression were calculated using a previously described method [72,73]. The ΔCt value described the difference between the threshold cycle (Ct) of the target gene and that of the reference *rpoD* gene. The ΔΔCt value described the difference between the ΔCt of *E*. *coli* WT and that of *E*. *coli* ZED. The difference in expression was calculated as 2^ΔΔCt^.

The frequency of the *ptsG* ^2.2^ allele in the end-point Z2 population was quantified by an established method [74] using the same real-time PCR supermix and instrument. Total DNA of this population and genomic DNA of evolved isolates Z2.2 and Z2.4 were extracted by DNeasy Blood & Tissue Kit (QIAGEN). Concentrations of DNA were determined by a Nanodrop ND-1000 (Thermo Scientific), and 30 ng of DNA was added to each real-time PCR reaction. To establish a standard curve, genomic DNA of Z2.2 and Z2.4 was mixed at defined ratios (0%, 5%, 10%, 20%, 40%, 60%, 100%) and quantified along with that of the Z2 population. The frequencies of the *ptsG* ^2.2^ allele were inversely correlated with the logarithm of Ct values of real-time PCR by the *ptsG* ^2.2^-specific primer pair HCEp126e/HCEp159.

### Quantification of expression profiles by fluorescent promoter reporters

GFP-based promoter reporter plasmids were generated previously [36,37] and introduced into *E*. *coli* through electroporation. The procedures for monitoring cell growth and GFP fluorescence were identical to those for growth profiling except that kanamycin (50 mg/l) was added to growth medium to prevent plasmid loss. In addition to OD, GFP readouts (excitation wavelength: 500 ± 5 nm, emission wavelength: 530 ± 10 nm) were also recorded at 10 min intervals. Transcriptional activity (defined as GFP/OD) and promoter activity (defined as dGPF/dt/OD [36]) were computed and plotted by MATLAB (MathWorks). Transcriptional activity and promoter activity at the exponential phase were quantified by averaging across the growth period corresponding to OD = 0.1–0.3. Expression profiles computed by these two equations yielded qualitatively similar results (Table 2, S3 Table).

### Determination of substrate secretion and uptake rates

Substrate uptake and secretion rates were determined during growth of *E*. *coli* in 40 ml of M9 medium supplemented with either glucose (3 g/l) or glucose (2 g/l) plus sodium acetate (2 g/l). Extracellular substrate and byproduct concentrations were measured by Agilent 1100 series HPLC stack in combination with an Aminex HPX-87H polymer column. Sugars were detected with a refractive index detector and organic acids with an UV/Vis detector. Substrate or product yields were calculated by linear regression of external concentration against biomass, and specific rates were calculated as yield multiplied by the growth rate. At least five time points during the exponential growth phase were used for the regression analysis.

### Determination of cAMP and redox cofactor concentrations

Three samples of exponentially growing cells at an OD between 0.45 and 0.6 were collected within a 15-min interval from 40 ml of M9 glucose (3 g/l) medium in a growth chamber kept at 37°C. For each sample, 2 ml of culture was vacuum filtered on a 0.45-μm pore size nitrocellulose filter (Millipore) and immediately washed with two volumes of fresh M9 glucose (3 g/l) medium. The filter was transferred into 4 ml of 60% (v/v) ethanol/water for extraction at 78°C for 2 min. Cell debris and nitrocellulose were removed by centrifugation at 14000 g at 4°C for 10 min. Metabolite extracts were dried at 0.12 mbar in a homemade speed vac set-up. Metabolite concentrations were determined by an ion-pairing ultrahigh performance liquid chromatography-tandem mass spectrometry method [75]. Dry metabolite extracts were resuspended in 100μl, 10μl of which was injected on a Waters Acquity UPLC with a Waters Acquity T3 end-capped reverse phase column (150 × 2.1 mm × 1.8μm; Waters Corporation, Milford, MA, USA). cAMP, NAD(H), and NADP(H) were detected on a tandem mass spectrometer (Thermo TSQ Quantum Triple Quadropole with Electron-Spray Ionization; Thermo Scientific, Waltham, MA, USA).

### Growth competition

Incubation conditions for growth competition were identical to those for evolution experiments. Evolved isolate Z2.2, its revertant Z2.2 *ptsG* ^WT^, and fluorescently labeled Z2.4 were first grown in 640 μl LB medium followed by one passage in 640 μl of M9 glucose (1 g/l) medium for physiological acclimation. Upon growth competition, Z2.2 and Z2.2 *ptsG* ^WT^ were mixed with fluorescently labeled Z2.4 at defined volume ratios (10%, 30%, 60%, 90%) and diluted 1:512 into 640 μl of fresh M9 glucose medium. Each day these mixed populations were diluted accordingly and grown in fresh growth medium. Changes in the ratios of non-fluorescent cells over 7 passages were monitored by a Cytek DxP8 flow cytometer for at least 45000 cell counts per sample.

## Supporting Information

S1 Fig Dynamics of adaptation of evolved populations. (A) Growth rates of the W populations. (B) Growth rates of the Z populations. (C) Correlation between the growth rates of end-point Z populations and the average growth rates of four Z isolates from each population. The red lines in (A) and (B) indicate the average growth rates of twelve replicate populations.
(EPS)

S2 Fig Sequence coverage of large indels in evolved isolates. (A) Two-fold amplification of the 139 kb *rhsB-rhsA* region in Z2.4. (B) Four-fold amplification of the 39 kb region including the *pntAB* operon in Z10.2. (C) Two-fold amplification of the 128 kb region including the *ptsA* gene in Z10.2. (D) Two-fold amplification of the 250 kb region between pseudogenes *'eptB* and *yifN'* in Z10.2. (E) Deletion of the 12 kb region between pseudogenes *'yaiT* and *yaiC'* in Z11.1.
(EPS)

S3 Fig mTH shows broad phylogenetic distribution. The presence of mTH genes (named as *pntAB* and *nnt* in prokaryotes and eukaryotes, respectively) in the genome sequences of 4073 distinct species at the finished or permanent draft status in GenBank (released before February 2014) was mapped to a tree of life published previously [76]. Branches that represent the three domains of life, bacteria (purple), eukaryotes (pink), and archaea (green) are color-coded. The abundance of mTH in each taxonomic branch is shown as a heat map and reported by Entrez Gene [77].
(EPS)

S4 Fig Changes in glucose and acetate concentrations during batch culture. (A) Growth in M9 medium supplemented with glucose (3 g/l). (B) Growth in M9 medium supplemented with glucose (2 g/l) and sodium acetate (2 g/l). Growth curves, glucose concentrations, and acetate concentrations are shown as black, blue, and red lines, respectively. Dotted lines indicate the depletion of glucose.
(EPS)

S5 Fig Growth responses of *E*. *coli* to acetate concentrations. Strains were grown in M9 glucose (1 g/l) medium supplemented with various amounts of acetate. The growth curve from one of the four replicate cultures is shown as the representative.
(EPS)

S1 Table Growth phenotypes of ancestral strains and evolved isolates. (DOC)

S2 Table Genotypic differences between *E*. *coli* MG1655 used in this study and the reference genome sequence (GenBank accession no. U00096.3). (DOC)

S3 Table Effects of adaptive mutations on promoter activity during the exponential phase. (DOC)

S4 Table Effects of adaptive mutations on redox cofactor concentrations. (DOC)

S5 Table List of plasmids. (DOC)

S6 Table List of primers and oligonucleotides. (DOC)
