# Supplementary material for: Transhydrogenase Promotes the Robustness and Evolvability of E. coli Deficient in NADPH Production
Source: PLoS Genet. 2015 Feb 25;11(2):e1005007. doi: 10.1371/journal.pgen.1005007 (PMC4340650; doi:10.1371/journal.pgen.1005007)
Supplement: S1 Table — (DOC) [file pgen.1005007.s006.doc]

**Table S1. Growth phenotypes of ancestral strains and evolved isolates.**

| Strains or isolates | Morphology^a^ | Growth rate^b^ | Yield^b,c^ | Diauxic growth |
| --- | --- | --- | --- | --- |
| *E. coli* WT | M | 0.664 ± 0.009 | 0.748 ± 0.015 | + |
| *E. coli* ZED | M | 0.566 ± 0.01 | 0.582 ± 0.018 | + |
| W1.1 | L | 0.949 ± 0.069 | 0.779 ± 0.048 | + |
| W1.2 | L | 0.952 ± 0.023 | 0.806 ± 0.029 | + |
| W1.3 | S | 0.94 ± 0.049 | 0.503 ± 0.032 | + |
| W1.4 | M | 0.933 ± 0.052 | 0.768 ± 0.035 | + |
| W2.1 | B | 1.001 ± 0.052 | 0.688 ± 0.041 | + |
| W2.2 | S | 0.957 ± 0.055 | 0.753 ± 0.015 | + |
| W2.3 | B | 0.977 ± 0.049 | 0.453 ± 0.04 | + |
| W2.4 | M | 0.976 ± 0.07 | 0.572 ± 0.029 | + |
| W3.1 | L | 0.921 ± 0.086 | 0.803 ± 0.017 | + |
| W3.2 | S | 0.93 ± 0.075 | 0.779 ± 0.054 | + |
| W3.3 | L | 0.95 ± 0.059 | 0.701 ± 0.056 | + |
| W3.4 | M | 0.884 ± 0.081 | 0.659 ± 0.054 | + |
| W4.1 | L | 0.949 ± 0.055 | 0.652 ± 0.034 | + |
| W4.2 | S | 0.866 ± 0.048 | 0.728 ± 0.026 | + |
| W4.3 | M | 0.869 ± 0.074 | 0.664 ± 0.041 | + |
| W4.4 | L | 0.893 ± 0.107 | 0.501 ± 0.034 | + |
| W5.1 | L | 0.889 ± 0.039 | 0.637 ± 0.036 | + |
| W5.2 | S | 0.902 ± 0.046 | 0.598 ± 0.02 | + |
| W5.3 | M | 0.935 ± 0.082 | 0.63 ± 0.025 | + |
| W5.4 | L | 0.96 ± 0.085 | 0.638 ± 0.045 | + |
| W6.1 | L, B | 0.947 ± 0.029 | 0.718 ± 0.013 | + |
| W6.2 | M | 0.673 ± 0.057 | 0.62 ± 0.029 | + |
| W6.3 | B | 0.963 ± 0.085 | 0.643 ± 0.044 | + |
| W6.4 | M | 0.903 ± 0.093 | 0.549 ± 0.012 | + |
| W7.1 | L | 0.929 ± 0.057 | 0.64 ± 0.031 | + |
| W7.2 | S | 0.903 ± 0.055 | 0.675 ± 0.043 | + |
| W7.3 | L | 0.949 ± 0.029 | 0.613 ± 0.026 | + |
| W7.4 | M | 0.966 ± 0.099 | 0.482 ± 0.02 | + |
| W8.1 | B | 0.926 ± 0.034 | 0.735 ± 0.028 | + |
| W8.2 | M | 0.94 ± 0.044 | 0.735 ± 0.02 | + |
| W8.3 | S, B | 0.933 ± 0.031 | 0.63 ± 0.022 | + |
| W8.4 | M | 0.925 ± 0.045 | 0.662 ± 0.021 | + |
| W9.1 | L | 0.925 ± 0.031 | 0.711 ± 0.028 | + |
| W9.2 | S | 0.975 ± 0.043 | 0.592 ± 0.031 | + |
| W9.3 | B | 0.959 ± 0.059 | 0.541 ± 0.047 | + |
| W9.4 | M | 0.895 ± 0.066 | 0.526 ± 0.045 | + |
| W10.1 | L | 0.894 ± 0.034 | 0.697 ± 0.028 | + |
| W10.2 | S | 0.725 ± 0.034 | 0.837 ± 0.02 | + |
| W10.3 | M | 0.959 ± 0.108 | 0.487 ± 0.007 | + |
| W10.4 | L | 0.89 ± 0.045 | 0.748 ± 0.035 | + |
| W11.1 | L | 0.969 ± 0.074 | 0.612 ± 0.026 | + |
| W11.2 | S | 0.936 ± 0.073 | 0.605 ± 0.025 | + |
| W11.3 | M | 0.979 ± 0.04 | 0.666 ± 0.03 | + |
| W11.4 | M | 0.974 ± 0.025 | 0.737 ± 0.029 | + |
| W12.1 | L | 1.021 ± 0.031 | 0.674 ± 0.025 | + |
| W12.2 | B | 0.941 ± 0.046 | 0.734 ± 0.048 | + |
| W12.3 | S | 0.664 ± 0.075 | 0.655 ± 0.036 | + |
| W12.4 | L | 1.016 ± 0.034 | 0.753 ± 0.049 | + |
| Z1.1 | B | 0.76 ± 0.015 | 0.752 ± 0.009 | + |
| Z1.2 | M | 0.816 ± 0.036 | 0.43 ± 0.021 | - |
| Z1.3 | S, B | 0.726 ± 0.021 | 0.673 ± 0.025 | + |
| Z1.4 | B | 0.836 ± 0.033 | 0.472 ± 0.036 | - |
| Z2.1 | L, B | 0.584 ± 0.049 | 0.544 ± 0.05 | - |
| Z2.2 | T | 0.554 ± 0.051 | 0.712 ± 0.035 | - |
| Z2.3 | M | 0.86 ± 0.045 | 0.513 ± 0.029 | + |
| Z2.4 | L, B | 0.895 ± 0.021 | 0.579 ± 0.012 | + |
| Z3.1 | L | 0.902 ± 0.069 | 0.669 ± 0.036 | + |
| Z3.2 | S | 0.842 ± 0.066 | 0.615 ± 0.027 | + |
| Z3.3 | B | 0.88 ± 0.057 | 0.48 ± 0.031 | + |
| Z3.4 | M | 0.837 ± 0.043 | 0.702 ± 0.016 | + |
| Z4.1 | L | 0.871 ± 0.023 | 0.65 ± 0.01 | + |
| Z4.2 | T | 0.524 ± 0.04 | 0.569 ± 0.014 | - |
| Z4.3 | S | 0.914 ± 0.094 | 0.694 ± 0.02 | + |
| Z4.4 | M | 0.954 ± 0.057 | 0.672 ± 0.027 | + |
| Z5.1 | B | 0.902 ± 0.044 | 0.716 ± 0.031 | + |
| Z5.2 | S | 0.92 ± 0.041 | 0.705 ± 0.038 | + |
| Z5.3 | L | 0.919 ± 0.043 | 0.728 ± 0.026 | + |
| Z5.4 | M | 0.743 ± 0.062 | 0.723 ± 0.024 | + |
| Z6.1 | B | 0.782 ± 0.036 | 0.722 ± 0.027 | + |
| Z6.2 | L | 0.799 ± 0.04 | 0.704 ± 0.015 | + |
| Z6.3 | S | 0.793 ± 0.036 | 0.779 ± 0.025 | + |
| Z6.4 | S | 0.721 ± 0.045 | 0.713 ± 0.045 | - |
| Z7.1 | L | 0.674 ± 0.081 | 0.625 ± 0.032 | - |
| Z7.2 | B | 0.727 ± 0.03 | 0.737 ± 0.009 | + |
| Z7.3 | S | 0.841 ± 0.042 | 0.665 ± 0.023 | - |
| Z7.4 | M | 0.72 ± 0.051 | 0.731 ± 0.008 | + |
| Z8.1 | L | 0.878 ± 0.053 | 0.834 ± 0.013 | + |
| Z8.2 | S | 0.853 ± 0.058 | 0.675 ± 0.047 | + |
| Z8.3 | M | 0.887 ± 0.05 | 0.79 ± 0.019 | + |
| Z8.4 | L | 0.915 ± 0.044 | 0.756 ± 0.034 | + |
| Z9.1 | B | 0.883 ± 0.044 | 0.517 ± 0.029 | + |
| Z9.2 | B | 0.909 ± 0.046 | 0.52 ± 0.016 | + |
| Z9.3 | S | 0.913 ± 0.086 | 0.73 ± 0.014 | + |
| Z9.4 | M | 0.896 ± 0.04 | 0.674 ± 0.027 | + |
| Z10.1 | M | 0.696 ± 0.049 | 0.54 ± 0.013 | + |
| Z10.2 | B | 1.006 ± 0.058 | 0.513 ± 0.023 | + |
| Z10.3 | S | 0.817 ± 0.067 | 0.661 ± 0.013 | + |
| Z10.4 | M | 0.997 ± 0.055 | 0.529 ± 0.023 | + |
| Z11.1 | B | 0.667 ± 0.044 | 0.793 ± 0.025 | - |
| Z11.2 | M | 0.652 ± 0.065 | 0.669 ± 0.031 | - |
| Z11.3 | L | 0.622 ± 0.03 | 0.67 ± 0.011 | - |
| Z11.4 | S | 0.619 ± 0.016 | 0.689 ± 0.011 | - |
| Z12.1 | L | 0.754 ± 0.027 | 0.741 ± 0.02 | + |
| Z12.2 | M | 0.89 ± 0.053 | 0.521 ± 0.021 | + |
| Z12.3 | S | 0.894 ± 0.022 | 0.524 ± 0.025 | + |
| Z12.4 | B | 0.799 ± 0.045 | 0.716 ± 0.017 | + |

^a^Morphology of colonies formed on M9 glucose agar after incubation at 37 °C for 3 days. L, big; M, medium; S, small; T, very small; B, blurry edge.

^b^Mean ± 95% C.I. of 6 independent measurements.

^c^Maximum OD values of growth curves were reported as a proxy for growth yield.
