# Supplementary material for: Transhydrogenase Promotes the Robustness and Evolvability of E. coli Deficient in NADPH Production
Source: PLoS Genet. 2015 Feb 25;11(2):e1005007. doi: 10.1371/journal.pgen.1005007 (PMC4340650; doi:10.1371/journal.pgen.1005007)
Supplement: S2 Table — (DOC) [file pgen.1005007.s007.doc]

**Table S2. Genotypic differences between *E. coli* MG1655 used in this study and the reference genome sequence (Genbank accession no. U00096.3)**.

| Gene or region | Genotypic difference | WT | ZED |
| --- | --- | --- | --- |
| *arpB* | H508N | + | + |
| *ddlA/iraP* | g→c (-307/-156) | + |  |
| *flhD* ^a^ | absence of IS*1H* upstream of *flhD* (-305/-306) in the reference sequence | + | + |
| *gltS/xanP* | t→c (-165/-115) | + | + |
| *hycA/hypA* | t→c (-63/-149) |  | + |
| *ppiC/yifN'* ^b^ | c→t (-121/+78) | + | + |
| *proY* | A235S | + | + |
| *scpC* | G197S | + | + |
| *sspA* | Y78S | + | + |
| *stfP-stfE* | 1.8 kb inversion due to homologous recombination between *stfP* and *stfE* | + | + |
| *ybjL/ybjM* | a→g (-104/-166) | + | + |
| *yeaR* | ::IS*2* (112/113) | + | + |
| *yhcC/gltB* | c→t (-437/-238) | + | + |
| *yjcO* | △2 bp (+55 - +56) | + | + |
| *ylbE* ^b^ | a→g (114) | + | + |
| *yncH* | A82V | + |  |
| *zwf-eda* | △4.3 kb |  | + |

^a^Also noted in Barker CS, Prüß BM, Matsumura P (2004) Increased motility of *Escherichia coli* by insertion sequence element integration into the regulatory region of the *flhD* operon. J. Bacteriol. 186: 7529–7537.

^b^Also noted in Freddolino PL, Amini S, Tavazoie S (2012) Newly identified genetic variations in common *Escherichia coli* MG1655 stock cultures. J. Bacteriol. 194: 303-306.
