# Supplementary material for: Transhydrogenase Promotes the Robustness and Evolvability of E. coli Deficient in NADPH Production
Source: PLoS Genet. 2015 Feb 25;11(2):e1005007. doi: 10.1371/journal.pgen.1005007 (PMC4340650; doi:10.1371/journal.pgen.1005007)
Supplement: S3 Table — (DOC) [file pgen.1005007.s008.doc]

**Table S3. Effects of adaptive mutations on promoter activity during the exponential phase.**

| Genotype | Promoter activity (dGFP/dt/OD)^a^ | | | | |
| --- | --- | --- | --- | --- | --- |
|  | *pntAB* | *acs* | *epd*-CBS | *epd* | CRP |
| WT | 1643 ± 314 | 139 ± 11 | 7120 ± 578 | 1160 ± 94 | 6.1 ± 0.1 |
| ZED | 2347 ± 136 | 142 ± 223 | 3512 ± 546 | 676 ± 176 | 5.4 ± 1.7 |
| ZED *pntAB* ^2.4^ | 2527 ± 243 | 100 ± 57 | 5545 ± 173 | 1177 ± 140 | 4.7 ± 0.5 |
| ZED *cyaA* ^8.4^ | 3304 ± 357 | 117 ± 156 | 3219 ± 377 | 1257 ± 372 | 2.6 ± 0.6 |
| ZED *cyaA* ^11.1^ | 3226 ± 66 | 347 ± 350 | 2853 ± 757 | 1200 ± 229 | 2.4 ± 0.2 |
| ZED *crp* ^11.1^ | 1418 ± 62 | 249 ± 62 | 16146 ± 694 | 1007 ± 102 | 16.1 ± 1.0 |
| ZED *ptsG* ^2.2^ | 1313 ± 247 | 278 ± 76 | 11450 ± 815 | 970 ± 42 | 11.8 ± 0.4 |
| ZED *ptsG* ^10.1^ | 1062 ± 192 | 351 ± 92 | 20316 ± 1487 | 1050 ± 50 | 19.3 ± 0.9 |
| ZED *ptsI* ^12.1^ | 2140 ± 144 | 57 ± 139 | 6839 ± 768 | 951 ± 186 | 7.3 ± 0.9 |

^a^Measurements are averaged across the range of OD = 0.1-0.3. Data are reported as means and 95% confidence intervals of averaged measurements from three replicate experiments.

^b^CRP activity is defined as the transcription driven by the *epd*-CBS hybrid promoter divided by that of the constitutive *epd* promoter.
