# Supplementary material for: Transhydrogenase Promotes the Robustness and Evolvability of E. coli Deficient in NADPH Production
Source: PLoS Genet. 2015 Feb 25;11(2):e1005007. doi: 10.1371/journal.pgen.1005007 (PMC4340650; doi:10.1371/journal.pgen.1005007)
Supplement: S4 Table — (DOC) [file pgen.1005007.s009.doc]

**Table S4. Effects of adaptive mutations on redox cofactor concentrations.**

| Genotype | NAD^+^ | NADH | NADP^+^ | NADPH |
| --- | --- | --- | --- | --- |
| *E. coli* WT | 891 ± 55 | 236 ± 44 | 228 ± 26 | 240 ± 35 |
| *E. coli* ZED | 962 ± 88 | 272 ± 45 | 253 ± 48 | 223 ± 20 |
| ZED *cyaA*^8.4^ | 790 ± 109 | 267 ± 61 | 186 ± 40 | 205 ± 39 |
| ZED *cyaA*^11.1^ | 821 ± 74 | 243 ± 29 | 216 ± 33 | 239 ± 47 |
| ZED *ptsG*^2.2^ | 893 ± 91 | 212 ± 53 | 234 ± 38 | 241 ± 46 |
| ZED *ptsG*^10.1^ | 910 ± 65 | 251 ± 46 | 225 ± 12 | 211 ± 25 |

Results are reported in **µM** as means ± 95% confidence intervals based on three independent measurements.
