# Supplementary material for: Transhydrogenase Promotes the Robustness and Evolvability of E. coli Deficient in NADPH Production
Source: PLoS Genet. 2015 Feb 25;11(2):e1005007. doi: 10.1371/journal.pgen.1005007 (PMC4340650; doi:10.1371/journal.pgen.1005007)
Supplement: S5 Table — (DOC) [file pgen.1005007.s010.doc]

**Table S5. List of plasmids.**

| Plasmid | Description | Source or reference |
| --- | --- | --- |
| pDS132 | *sacB*-based suicide plasmid for allelic exchange; ^a^Cm^r^ | Philippe, N. et al (2004) |
| pHC08 | *loxP-t_rrnB_-P_tacA_-mCherry-t_T7_*; ^b^Tc^r^ | Lee et al (2009) |
| pHC140 | pDS132 with a polylinker; Cm^r^ | This study |
| pHC145 | pHC140 with a synthetic Δ*pntAB* allele; Cm^r^ | This study |
| pHC150e | pHC140 with the *cyaA*^8.4^ allele; Cm^r^ | This study |
| pHC151w | pHC140 with the *ptsG*^WT^ allele (with respect to *ptsG*^10.1^); Cm^r^ | This study |
| pHC151e | pHC140 with the *ptsG*^10.1^ allele; Cm^r^ | This study |
| pHC152e | pHC140 with the *ptsI*^12.1^ allele; Cm^r^ | This study |
| pHC153w | pHC140 with the *ptsG*^WT^ allele (with respect to *ptsG*^2.2^); Cm^r^ | This study |
| pHC153e | pHC140 with the *ptsG*^2.2^ allele; Cm^r^ | This study |
| pHC154e | pHC140 with the *pntAB*^2.4^ allele; Cm^r^ | This study |
| pHC155e | pHC140 with the *cyaA*^11.1^ allele; Cm^r^ | This study |
| pHC156e | pHC140 with the *crp*^11.1^ allele; Cm^r^ | This study |
| pHC161m | pHC140 with the *t_rrnB_-P_tacA_-mCherry-t_T7_* cassette from pHC08; Cm^r^ | This study |
| pHC175 | pHC161m with the downstream region of the *araBAD* operon; Cm^r^ | This study |
| pHC176 | pHC175 with the upstream region of the *araBAD* operon; Cm^r^ | This study |
| pHC179 | pHC176 with *P_tacA_* replaced by the *P_A1_* promoter; Cm^r^ | This study |

^a^Cm^r^, chloramphenicol resistance

^b^Tc^r^, tetracycline resistance.
