# Supplementary material for: Transhydrogenase Promotes the Robustness and Evolvability of E. coli Deficient in NADPH Production
Source: PLoS Genet. 2015 Feb 25;11(2):e1005007. doi: 10.1371/journal.pgen.1005007 (PMC4340650; doi:10.1371/journal.pgen.1005007)
Supplement: S6 Table — (DOC) [file pgen.1005007.s011.doc]

**Table S6. List of primers and oligonucleotides.**

| Name | Sequence | Application |
| --- | --- | --- |
| linker.F | 5'-CGAGATCTTAAGCATGCTAGCATATGACTAGTGGGCCCTGCA-3' | Cloning pHC140 |
| linker.R | 5'-gggcccactagtcatatgctagcatgcttaagatctcgagct-3' | Cloning pHC140 |
| HC140p1 | 5'-GCATAAAGTTGCCTTTTTAATCACA-3' | Sequencing pHC140 |
| HC140p2 | 5'-cacttaacggctgacatgg-3' | Sequencing pHC140 |
| HCEp15 | 5'-AAGACGGGATCAACCAGGTTCAATG-3' | *rpoD* qPCR |
| HCEp16 | 5'-GTCTTCATCTTCGTCATCGTCCAGA-3' | *rpoD* qPCR |
| HCEp19 | 5'-CACAATCGCTGGACGCACTAAGC-3' | *pntAB* qPCR |
| HCEp20 | 5'-GTGTCGAATGCACGCACAATCG-3' | *pntAB* qPCR |
| HCEp64A | 5'-AGCTAGCCAGTTACCGCTGTTAC-3' | Amplifying *pntAB* upstream |
| HCEp65 | 5'-ccgtcaggggatattcccttccatcggtttta-3' | Amplifying *pntAB* upstream |
| HCEp66 | 5'-AGGGAATATCCCCTGACGGCCTCTGCT-3' | Amplifying *pntAB* downstream |
| HCEp67 | 5'-ACTCGAGtatcgcaactctattatgacgc-3' | Amplifying *pntAB* downstream |
| HCEp68 | 5'-CATCGGCTTTATAGAGGTCAG-3' | Detecting Δ*pntAB* |
| HCEp69 | 5'-cgctcaacgagttgacgc-3' | Detecting Δ*pntAB* |
| HCEp111 | 5'-ACTCGAGACTATCACCATCCGCTAATGC-3' | Cloning pHC150e |
| HCEp112 | 5'-AGCTAGCAGATTCGGTTCCGAGAGATC-3' | Cloning pHC150e |
| HCEp113 | 5'-CAGTCTACTGCCGACATTGTTGC-3' | Detecting c*yaA* |
| HCEp114w | 5'-GAGCTGCCAAAGGCTGG-3' | Detecting *cyaA*^WT^ allele |
| HCEp114e | 5'-GAGCTGCCAAAGGCTGA-3' | Detecting *cyaA*^8.4^ allele |
| HCEp115 | 5'-AGCTAGCGTGACGTTGCAGAATACATG-3' | Cloning pHC151we |
| HCEp116 | 5'-ACTCGAGCAGAGTGCCAGATAGCAA-3' | Cloning pHC151we |
| HCEp117 | 5'-GGAAAAGAGAATCAACCTGCGATGG-3' | Detecting *ptsG* |
| HCEp118w | 5'-CGAGGGCGACACCGATC-3' | Detecting *ptsG*^WT^ allele |
| HCEp118e | 5'-AAAGCCGAGGGCGACAAAATC-3' | Detecting *ptsG*^10.1^ allele |
| HCEp119 | 5'-ACTCGAGCTGCTCATGAAGTTATCGAAG-3' | Cloning pHC152e |
| HCEp120 | 5'-AGCTAGCACACATGCCAGTCCATTTG-3' | Cloning pHC152e |
| HCEp121 | 5'-CATCGCCAGCAAGCTCAC-3' | Detecting *ptsI* |
| HCEp122w | 5'-TGAGTTCCTGTTCATGGACC-3' | Detecting *ptsI*^WT^ allele |
| HCEp122e | 5'-CTGAGTTCCTGTTCATGGACT-3' | Detecting *ptsI*^12.1^ allele |
| HCEp123 | 5'-ACTCGAGCCACTGGTACTGCATTTAC-3' | Cloning pHC153we |
| HCEp124 | 5'-AGCTAGCAAAAGGCAGCCATCTGG-3' | Cloning pHC153we |
| HCEp125 | 5'-GCAGTTGTTGCCTATGGCATCATG-3' | Detecting *ptsG* |
| HCEp126w | 5'-CGATCGGCTCGGTGATAC-3' | Detecting *ptsG*^WT^ allele |
| HCEp126e | 5'-TCGATCGGCTCGGTGATAA-3' | Detecting *ptsG*^2.2^ allele |
| HCEp127 | 5'-AGCTAGCGCCGAGACTGTTTGCTG-3' | Cloning pHC154e |
| HCEp128 | 5'-ACTCGAGGAGAATGCCATAGCAGAAAGC-3' | Cloning pHC154e |
| HCEp129 | 5'-GGTGTCAGTTCTGTTGCTGC-3' | Detecting *pntAB* |
| HCEp130w | 5'-GTATGCCAATTCGCATGATATT-3' | Detecting *pntAB*^WT^ allele |
| HCEp130e | 5'-GTATGCCAATTCGCATGATATC-3' | Detecting *pntAB*^2.4^ allele |
| HCEp131 | 5'-ACTCGAGGCAAAGTTGTTCCTCTGAC-3' | Cloning pHC155e |
| HCEp132 | 5'-AGCTAGCCACCAGTTTATTCAGGTAACG-3' | Cloning pHC155e |
| HCEp133 | 5'-GCCGTTAAACCATGCCCAC-3' | Detecting c*yaA* |
| HCEp134w | 5'-GATTGTATCGTTTGGTCTCGAT-3' | Detecting *cyaA*^WT^ allele |
| HCEp134e | 5'-GATTGTATCGTTTGGTCTCGAA-3' | Detecting *cyaA*^11.1^ allele |
| HCEp135 | 5'-ACTCGAGAATGTGCGTAAACAGGCGTG-3' | Cloning pHC156e |
| HCEp136 | 5'-AGCTAGCCACTCCGACGGGATTAAC-3' | Cloning pHC156e |
| HCEp137 | 5'-CGCCACATCGGGGGAAAC-3' | Detecting c*rp* |
| HCEp138w | 5'-AATGGTTCTTGTCTCATTGCC-3' | Detecting *crp*^WT^ allele |
| HCEp138e | 5'-AATGGTTCTTGTCTCATTGCT-3' | Detecting *crp*^11.1^ allele |
| HCEp159 | 5'-ACTGTCTGGTGGCTTCCTGTTCAA-3' | *ptsG*^2.2^ qPCR |
| HC161p1 | 5'-AAGCATGCTAGCATATGCCAGGCATCAAATAAAACG-3' | Cloning pHC161m |
| HC161p2 | 5'-AAACTAGTGCACGGATATAGTTCCTCCTTTCAGC-3' | Cloning pHC161m |
| HCEp161 | 5'-AAGCATGCTAGCATATGCCAGGCATCAAATAAAACG-3' | Amplifying *araD* downstream |
| HCEp162 | 5'-AAACTAGTGCACGGATATAGTTCCTCCTTTCAGC-3' | Amplifying *araD* downstream |
| HCEp163 | 5'-TTGGGCCCTACTGCCCGTAATATGCCTTCG-3' | Amplifying *araB* upstream |
| HCEp164 | 5'-TTACTAGTCAACCTGGTCACGCTCAC-3' | Amplifying *araB* upstream |
| HCEp177 | 5'-CACTATCACGGCTGAATCGT-3' | Sequencing *pntAB* |
| HCEp178 | 5'-GCTGTAACTAATCCTCCAGACA-3' | Sequencing *pntAB* |
| HCEp179 | 5'-GGTGCTTATAGCCAGCATT-3' | Sequencing *pntAB* |
| HCEp180 | 5'-TGCTTTCCGTGCCGTTGA-3' | Sequencing *pntAB* |
| HCEp181 | 5'-GCGAAAAGTGGTAACGGT-3' | Sequencing *cyaA* |
| HCEp182 | 5'-GAAATCGAAATGCACCACCTG-3' | Sequencing *cyaA* |
| HCEp183 | 5'-CTCTACAGCCCGTGTGAGA-3' | Sequencing *cyaA* |
| HCEp184 | 5'-CGGGTGAAACAGTCAGTTTC-3' | Sequencing *cyaA* |
| HCEp185 | 5'-TCGATGTCACCTATCCTTAGAG-3' | Sequencing *pykF* |
| HCEp186 | 5'-GGGAAGGCACTTATTTTTGATC-3' | Sequencing *pykF* |
| PA1.F | 5'-CGCGTTATCAAAAAGAGTATTGACTTAAAGTCTAACCTATAGGATACTTACAGCCATCGAGAGGGAT-3' | *P_A1_* promoter for pHC179 |
| PA1.R | 5'-TAATCCCTCTCGATGGCTGTAAGTATCCTATAGGTTAGACTTTAAGTCAATACTCTTTTTGATAA-3' | *P_A1_* promoter for pHC179 |
